# Genomewide characterisation of the genetic diversity of carotenogenesis in bacteria of the order *Sphingomonadales*

**DOI:** 10.1099/mgen.0.000172

**Published:** 2018-04-05

**Authors:** Shivakumara Siddaramappa, Vandana Viswanathan, Saravanamuthu Thiyagarajan, Anushree Narjala

**Affiliations:** ^1^​ Institute of Bioinformatics and Applied Biotechnology, Biotech Park, Electronic City, Bengaluru 560100, Karnataka, India; ^2^​ Manipal Academy of Higher Education, Manipal 576104, Karnataka, India

**Keywords:** *Sphingomonadales*, *Sphingomonadaceae*, *Erythrobacteraceae*, carotenogenesis, LOG, DUF2141

## Abstract

The order *Sphingomonadales* is a taxon of bacteria with a variety of physiological features and carotenoid pigments. Some of the coloured strains within this order are known to be aerobic anoxygenic phototrophs that contain characteristic photosynthesis gene clusters (PGCs). Previous work has shown that majority of the ORFs putatively involved in the biosynthesis of C_40_ carotenoids are located outside the PGCs in these strains. The main purpose of this study was to understand the genetic basis for the various colour/carotenoid phenotypes of the strains of *Sphingomonadales*. Comparative analyses of the genomes of 41 strains of this order revealed that there were different patterns of clustering of carotenoid biosynthesis (*crt*) ORFs, with four ORF clusters being the most common. The analyses also revealed that co-occurrence of *crtY* and *crtI* is an evolutionarily conserved feature in *Sphingomonadales* and other carotenogenic bacteria. The comparisons facilitated the categorisation of bacteria of this order into four groups based on the presence of different *crt* ORFs. Yellow coloured strains most likely accumulate nostoxanthin, and contain six ORFs (group I: *crtE*, *crtB*, *crtI*, *crtY*, *crtZ*, *crtG*). Orange coloured strains may produce adonixanthin, astaxanthin, canthaxanthin and erythroxanthin, and contain seven ORFs (group II: *crtE*, *crtB*, *crtI*, *crtY*, *crtZ*, *crtG*, *crtW*). Red coloured strains may accumulate astaxanthin, and contain six ORFs (group III: *crtE*, *crtB*, *crtI*, *crtY*, *crtZ*, *crtW*). Non-pigmented strains may contain a smaller subset of *crt* ORFs, and thus fail to produce any carotenoids (group IV). The functions of many of these ORFs remain to be characterised.

## Data Summary

A full list of accession numbers for the whole-genome sequence data and locus tags of protein sequences used in this study are provided in Tables 1 and S1 (available with the online version of this article), respectively. Additional data is provided in Table S2 and Fig. S1.

**Table 1.** Features of the strains of *Sphingomonadales* and their genomes used in this study

Serial no.	Species (strain)	Chromosome size (bp) [genome status]	G+C (mol%)	GenBank/RefSeq accession no.	*crt* ORFs identified	Group based on *crt* genotype	Colour of the strain	Carotenoids produced	Reference (for colour and/or carotenoid)*
1	*Sphingopyxis alaskensis* (RB2256^T^)	3345170 [complete]	65.50	CP000356	*E*, *B*, *I*, *Y*, *Z*, *G*	I	Yellow to beige	Nostoxanthin^P^, caloxanthin^P^	[77]
2	*Sphingopyxis macrogoltabida* (EY-1)	4757879 [complete]	64.90	CP012700	*E*, *B*, *I*, *Y*, *Z*, *G*	I	Unknown	Unknown	None
3	*Sphingopyxis fribergensis* (Kp5.2^T^)	4993584 [complete]	63.90	CP009122	*E*, *B*, *I*, *Y*, *Z*, *G*	I	Yellow	Nostoxanthin^P^, caloxanthin^P^	[78]
4	*Sphingomonas sanxanigenens* (DSM 19645^T^)	6205897^PGC^[complete]	66.80	CP006644	*E*, *B*, *I*, *Y*, *Z*, *G*	I	Colourless/white	Phytoene/none^P^	[64]
5	*Sphingorhabdus* sp. (M41)	3339521 [complete]	56.70	CP014545	*E, B, I, Y, Z, G*	I	Colourless/white	Phytoene/none^P^	Personal communication^1^
6	*Sphingopyxis* sp. (113P3)	4420776 [complete]	64.00	CP009452	*E, B, I, Y, Z, G*	I	Yellowish brown	Nostoxanthin^P^, caloxanthin^P^	[79]
7	*Sphingopyxis terrae* (NBRC 15098^T^)	3979087 [complete]	64.60	CP013342	*E, B*, *I*, *Y*, *Z*, *G*	I	Light or deep-yellow	Nostoxanthin^P^, caloxanthin^P^	[80]
8	*Sphingobium* sp. (SYK-6)	4199332 [complete]	65.60	NC_015976	*E*, *B*, *I*, *Y*, *Z*, *G*	I	Yellow	Nostoxanthin^P^, caloxanthin^P^	Personal communication^2^
9	*Sphingomonas hengshuiensis* (WHSC-8^T^)	5191536^PGC^ [complete]	66.70	CP010836	*E*, *B*, *I*, *Y*, *Z*, *G*	I	Yellow	Nostoxanthin^P^, caloxanthin^P^	[65]
10	*Sphingomonas* sp. (Root241)	4212322 [draft]	66.00	NZ_LMIV00000000	*E*, *B*, *I*, *Y*, *Z*, *G*	I	Unknown	Unknown	None
11	*Sphingomonas* sp. (ATCC 31555)	4046117 [draft]	65.90	NZ_ALBQ00000000	*E*, *B*, *I*, *Y*, *Z*, *G*, *W*	II	Red	Canthaxanthin and others^P^	[81]
12	*Sphingomonas paucimobilis* (NBRC 13935^T^)	4327402 [draft]	65.70	NZ_BBJS00000000	*E*, *B*, *I*, *Y*, *Z*, *G*	I	Yellow	Nostoxanthin^C^, caloxanthin^P^	[82]
13	*Sphingomonas astaxanthinifaciens* (DSM 22298^T^)	2533034^PGC^ [draft]	68.40	NZ_JONN00000000	*E*, *B*, *I*, *Y*, *Z*, *W*, *X*	III	Red	Astaxanthin and its derivatives^C^	[58]
14	*Sphingobium chlorophenolicum* (L-1)	3080818 [chromosome 1, complete]	63.90	CP002798	*E*, *B*, *I*, *Y*, *Z*, *G*	I	Yellow	Nostoxanthin^P^, caloxanthin^P^	[83]
15	*Sphingobium* sp. (MI1205)	3351250 [chromosome 1, complete]	62.30	CP005188	*E*, *B*, *I*, *Y*, *Z*, *G*	I	Brownish–yellow	Nostoxanthin^P^, caloxanthin^P^	[84]
16	*Sphingobium* sp. (EP60837)	2669660 [chromosome 1, complete]	62.40	CP015986	*E*, *B*, *I*, *Y*, *Z*, *G*	I	Colourless/white	Phytoene/none^P^	Personal communication^3^
17	*Sphingobium* sp. (YBL2)	4766421 [complete]	64.80	CP010954	*E*, *B*, *I*, *Y*, *Z*, *G*	I	Yellow	Nostoxanthin^P^, caloxanthin^P^	[85]
18	*Novosphingobium aromaticivorans* (DSM 12444^T^)	3561584 [complete]	65.20	CP000248	*E*, *B*, *I*, *Y*, *Z*, *G*	I	Yellow	Nostoxanthin^P^, caloxanthin^P^	[86]
19	*Citromicrobium* sp. (JL477)	3258499^PGC^ [complete]	65.00	CP011344	*E*, *B*, *I*, *Y*, *Z*, *G*	I	Yellow	Nostoxanthin^P^, caloxanthin^P^	Personal communication^4^
20	*Altererythrobacter dongtanensis* (KCTC 22672^T^)	3009495 [complete]	65.80	CP016591	*E*, *B*, *I*, *Y*, *Z*, *G*	I	Yellow	Nostoxanthin^P^, caloxanthin^P^	[87]
21	*Altererythrobacter namhicola* (JCM 16345^T^)	2591679 [complete]	65.00	CP016545	*E*, *B*, *I*, *Y*, *Z*, *G*, *W*	II	Orange	Canthaxanthin and others^P^	[88]
22	*Altererythrobacter epoxidivorans* (CGMCC 1.7731^T^)	2786256 [complete]	61.50	CP012669	*E*, *B*, *I*, *Y*, *Z*, *G*	I	Yellow	Nostoxanthin^P^, caloxanthin^P^	[89]
23	*Altererythrobacter atlanticus* (26DY36^T^)	3386291 [complete]	61.90	CP011452	*E*, *B*, *I*, *Y*, *Z*, *G*	I	Yellow	Nostoxanthin^P^, caloxanthin^P^	[90]
24	*Altererythrobacter ishigakiensis* (NBRC 107699^T^)	2673978 ^PGC^[complete]	56.90	CP015963	*E*, *B*, *I*, *Y*, *Z*, *G*, *W*	II	Orange–red	Canthaxanthin and others^C^	[63]
25	*Erythrobacter litoralis* (HTCC2594)	3052398 [complete]	63.10	CP000157	*E*, *B*, *I*, *Y*, *Z*, *G*, *W*	II	Pink	Unknown	[91]
26	*Erythrobacter atlanticus* (s21-N3^T^)	3012400 [complete]	58.20	CP011310	*E*, *B*, *I*, *Y*, *Z*, *G*	I	Yellow–brown	Nostoxanthin^P^, caloxanthin^P^	[92]
27	*Sphingobium yanoikuyae* (ATCC 51230^T^)	5500358 [draft)]	64.40	NZ_AGZU00000000	*E*, *B*, *I*, *Y*, *Z*, *G*	I	Creamy white	Phytoene/none^P^	[82]
28	*Novosphingobium* sp. (PP1Y)	3911486 [complete]	63.70	FR856862	*E*, *B*, *I*, *Y*, *Z*, *G*	I	Yellow	Nostoxanthin^P^, caloxanthin^P^	[93]
29	*Novosphingobium pentaromativorans* (US6-1^T^)	3979506 [complete]	63.50	CP009291	*E*, *B*, *I*, *Y*, *Z*, *G*	I	Yellow	Nostoxanthin^P^, caloxanthin^P^	[94]
30	*Porphyrobacter neustonensis* (DSM 9434^T^)	3090363^PGC^[complete]	65.30	CP016033	*E*, *B*, *I*, *Y*, *Z*, *G*, *W*	II	Orange	Canthaxanthin and others^P^	[10]
31	*Sphingomonas* sp. (NIC1)	3408545 [complete]	67.40	CP015521	*E*, *B*, *I*, *Y*, *Z*, *G*	I	Unknown	Unknown	None
32	*Sphingomonas melonis* (TY)	4100783 [draft]	67.10	NZ_LQCK00000000	*E*, *B*, *I*, *Y*, *Z*, *G*	I	Yellow	Nostoxanthin^P^, caloxanthin^P^	[95]
33	*Altererythrobacter marensis* (KCTC 22370^T^)	2885033 [complete]	64.70	CP011805	*E*, *B*, *I*, *Y*, *Z*, *G*	I	Yellow	Nostoxanthin^P^, caloxanthin^P^	[96]
34	*Croceicoccus naphthovorans* (PQ-2^T^)	3543806 [complete]	62.60	CP011770	*E*, *B*, *I*, *Y*, *Z*, *G*	I	Yellow	Nostoxanthin^P^, caloxanthin^P^	[97]
35	*Sphingomonas taxi* (ATCC 55669)	3859099 [complete]	68.00	CP009571	*E*, *B*, *I*, *Y*, *Z*, *G*, *W*	II	Yellow to orange	Canthaxanthin and others^P^	[98]
36	*Sphingomonas* sp. (RIT328)	4343511 [draft]	68.30	NZ_JFYV00000000	*E*, *B*, *I*, *Y*, *Z*, *G*, *W*	II	Unknown	Unknown	None
37	*Sphingobium japonicum* (UT26S)	3514822 [chromosome 1, complete]	64.80	NC_014006	*E*, *B*, *I*, *Y*, *Z*, *G*	I	Yellow	Nostoxanthin^P^, caloxanthin^P^	[99]
38	*Sphingomonas* sp. (MM-1)	4054833 [complete]	67.20	CP004036	*E*, *I*, *Y*	IV	Colourless/white	None	Personal communication^5^
39	*Sphingobium baderi* (DE-13)	4107398 [complete]	62.40	CP013264	*E*, *I*	IV	Colourless/white	None	[100]
40	*Sphingomonas wittichii* (RW1^T^)	5382261 [complete]	68.40	CP000699	*E*, *Y*	IV	Greyish-white (faintly yellow)	None	[101]
41	*Sphingopyxis granuli* (TFA)	4679853 [complete]	66.20	CP012199	*E*	IV	Yellowish	None	[102]

C, Confirmed in one or more studies published previously; P, presumptive based on the colour of the strain reported and the *crt* ORFs identified; PGC, genome contains a putative photosynthesis gene cluster – production of spirilloxanthin (using CrtC, CrtD and CrtF) and Bchl *a* from this cluster can potentially affect the colour of the host strains; T, type strain.

1, Dr Che Ok Jeon, Chung-Ang University, Republic of Korea; 2, Dr Eiji Masai, Nagaoka University of Technology, Japan; 3, Dr Byung-Yong Kim, ChunLab, Inc., Republic of Korea; 4, Dr Qiang Zheng, Xiamen University, People's Republic of China; 5, Dr Yuji Nagata, Tohoku University, Japan.

**Impact Statement**

Despite pigmentation being the most obvious phenotype among members of the *Sphingomonadales*, not much is known about the genetic basis of its biosynthesis across different species and genera. The present study sought to fill this gap by leveraging the wealth of genomic data that has become available in the last 5 years. Through in-depth analyses of the genomes of 41 strains of *Sphingomonadales*, the genotypes (based on a combination of *crtE*, *crtB*, *crtI*, *crtY*, *crtZ*, *crtW* and/or *crtG*) that could engender the various colour/carotenoid phenotypes have been identified. This cataloguing effort would benefit not only bacteriologists interested in systematics, but also biologists investigating carotenoid biosynthesis and its evolution in different organisms. Although it is well known that carotenoid and cytokinin biosynthesis requires precursors derived from isoprenoids, the genetic association between these two pathways had not been identified hitherto. In this context, the discovery within *crt* loci of an ORF encoding a putative homologue of LOG has opened up novel frontiers in basic as well as applied research.

## Introduction

The order *Sphingomonadales* was circumscribed within the hierarchical system of the second edition of the *Bergey's Manual of Systematic Bacteriology* as a distinct phylogenetic branch of the *Alphaproteobacteria*, which was a novel class of the phylum *Proteobacteria* [1–3]. Currently, *Sphingomonadaceae* and *Erythrobacteraceae* are the only two families within this order [4]. The class *Alphaproteobacteria* contains many well-characterised phototrophs, including the Bchl *a*-producing aerobic anoxygenic phototrophs (AAPs), which are believed to be incapable of using light as the sole source of energy [5, 6]. Thus, most of the AAPs are photoheterotrophs and inhabit a variety of nutrient-rich aquatic and terrestrial environments [6, 7]. It has been postulated that AAPs are descendants of anaerobic purple bacteria, and studies of these organisms may provide clues to understanding the evolution of non-photosynthetic aerobes [6]. Historically, *Erythrobacter longus* strain OCh101 is among the well-known AAPs [8, 9], and is incidentally a member of the *Sphingomonadales* [4]. Nevertheless, AAPs appear to be a minority within this order, because only a few species/strains containing Bchl *a* have been identified in the last four decades [10–13]. Comparative genomic analyses have shown that the photosynthesis gene clusters (PGCs) are conserved in *Erythrobacter* sp. NAP1 and *Citromicrobium* sp. JL354 [14]. A similar cluster has also been identified in the genome of *Porphyrobacter neustonensis* DSM 9434 [15]. These clusters contain ORFs encoding putative proteins of the photosynthetic reaction centre and light harvesting complexes. Not surprisingly, some ORFs within these PGCs were predicted to be involved in the biosynthesis of Bchl *a* [14, 15].

A feature that is more obvious and common than anoxygenic phototrophy, but equally fascinating, among members of the *Sphingomonadales* is their pigmentation [3]. While providing the description of *Sphingomonadaceae* fam. nov., Kosako *et al.* [11] reported the different colours (e.g. yellow, orange and brown) of the species. The description of *Erythrobacteraceae* fam. nov. also noted that the members produced yellow, orange and pink pigments [4]. Therefore, it appears that being ‘coloured’ is a common phenotypic feature among members of these two families, and the ‘non-coloured’ strains (e.g. *Zymomonas* spp.) are most likely an exception to the rule. The vivid colours are known to be due to different carotenoids [16, 17], some of which have been well documented in the Prokaryotic Carotenoid Database [18] and the Carotenoids Database [19]. Although the biosynthesis of carotenoid pigments by photoautotrophs is a physiological necessity [20], their presence in AAPs and non-photosynthetic aerobes is somewhat of a mystery. It has been proposed that carotenoid pigments in these bacteria may be involved in protecting the cell from reactive oxygen species and high-energy radiation [7, 20]. It has also been reported that the carotenoid compositions of anaerobic purple bacteria are different from those of AAPs, and that AAPs of the order *Sphingomonadales* contain a variety of carotenoids, including β-carotene, zeaxanthin, caloxanthin, nostoxanthin, spirilloxanthin, spheroidene, erythroxanthin sulfate and caloxanthin sulfate [20]. Interestingly, most of the ORFs putatively involved in the biosynthesis of carotenoids were located outside the PGCs in the genomes of AAPs of the order *Sphingomonadales* [14, 15]. Functional genomic studies have provided valuable insights into carotenogenesis in members of the *Sphingomonadaceae*, and have identified ORFs involved in the biosynthesis of various carotenoids [21–23]. The objectives of this study were to extend the current knowledge about carotenogenesis from a few species to several genera within the order *Sphingomonadales*, and to identify novel genes that may have a role in carotenoid biosynthesis or modification among these bacteria.

## Methods

### Selection of bacterial strains

Bacterial strains included in this study were carefully selected so that type strains representing the major genera and species of the two families of the order *Sphingomonadales* made up at least 50 % of the pigmented cohort. This selection ensured that an authentic publication/reference for the colour/phenotype would be available. Other strains were selected based on the availability of their genome sequences in GenBank. Among these ‘other strains’, the majority had information about their colour/phenotype (either in the form of a publication or through personal communication). The final list included 41 strains, of which 34 had their genomes completely sequenced (Table 1). These strains represented the genera *Sphingomonas* (12), *Sphingobium* (8), *Sphingopyxis* (6), *Altererythrobacter* (6), *Novosphingobium* (3), *Erythrobacter* (2), *Citromicrobium* (1), *Croceicoccus* (1), *Porphyrobacter* (1) and *Sphingorhabdus* (1). Two of the strains (JL477 and DSM 9434) had already been confirmed to contain PGCs [15, 24].

### Genome annotation and identification of orthologues

The genome sequences of the 41 strains were retrieved from GenBank and annotated using the rast (rapid annotation using subsystem technology) server (http://rast.nmpdr.org/). The *crt* ORFs within these annotated genomes were identified by blastp analyses using the protein sequences from *Sphingomonas elodea* strain ATCC 31461 [21] as queries. The PGCs within these genomes were identified using protein sequences from the PGC of *P. neustonensis* DSM 9434 [15] as queries. The ORFs were deemed orthologous if the putative proteins encoded by them had at least 30 % identity and less than 20 % difference in length (with a bit score of at least 50 and an *E* value <10^−10^) during blastp analyses. The order and orientation of the *crt* ORFs in complete genomes were checked using the sequence-based comparison tool available within the SEED Viewer. The synteny of ORFs (i.e. the occurrence of ORFs in the same order and orientation within a locus) was also an important criterion in the identification of orthologues. In strains/genomes where more than one potential orthologue was identified for a given *crt* ORF, the orthologue with the highest percent identity was chosen. The locations of the *crt* loci/ORFs within the respective chromosomes were assessed using the ‘Feature Table’ option of the SEED Viewer. Overlapping ORFs were checked and confirmed by manual curation of their sequences. Raw maps of the *crt* loci were copied from SEED Viewer and edited/refined using Microsoft Paint.

### Analyses of protein sequences

Almost all protein sequences were retrieved from GenBank and identified using their standard locus tags. For some strains, the sequences had to be derived from their genome annotations within rast and assigned convenient locus tags. A preliminary alignment of the orthologous protein sequences was generated using Clustal Omega (http://www.ebi.ac.uk/Tools/msa/clustalo/) with default parameters. Orthologues showing misalignment were excluded from further analyses. Clustal Omega alignment was also crucial to predict the length of the proteins and assign start codons (ATG, GTG, TTG or CTG in that preferential order). Manual trimming (or ‘extension’) of ORFs that appeared to be incorrectly annotated in GenBank or rast was performed based on this alignment. ClustalW (http://embnet.vital-it.ch/software/ClustalW.html) was the tool of choice to obtain a ‘pir’ output of the protein sequences. The boxshade server (http://www.ch. embnet.org/software/BOX_form.html) was used to further align the ‘pir’ output from ClustalW and visualise the conserved positions within the protein sequences.

### Protein secondary structure prediction and phylogenetic analyses

Homologous protein sequences were identified using blastp analysis and acquired from GenBank, rast or the Protein Data Bank (PDB). Protein homologues with known crystal structures were used for predicting the secondary structure of the query. Multiple sequence alignment of the protein homologues was performed using Clustal Omega. The aligned sequences were analysed using ESPript (http://espript.ibcp.fr/ESPript/ESPript/) to depict similarities and secondary structure information. For phylogenetic analyses, relevant homologues (showing at least 40 % identity) from bacteria outside the order *Sphingomonadales* were included in the dataset when necessary. One of the datasets contained 31 sequences, whereas the other had just 7. Sequences were aligned using ClustalW and evolutionary distances were computed by analyses using the Molecular Evolutionary Genetics Analysis (mega 7.0) software package. Phylogeny was reconstructed using the maximum-likelihood method with the JTT or LG+F substitution model. Reliability of the trees was estimated using bootstrap methods (minimum of 500 replicates).

## Results and Discussion

### Clustering patterns of *crt* ORFs in strains of *Sphingomonadales* are variable

**Fig. 1.**
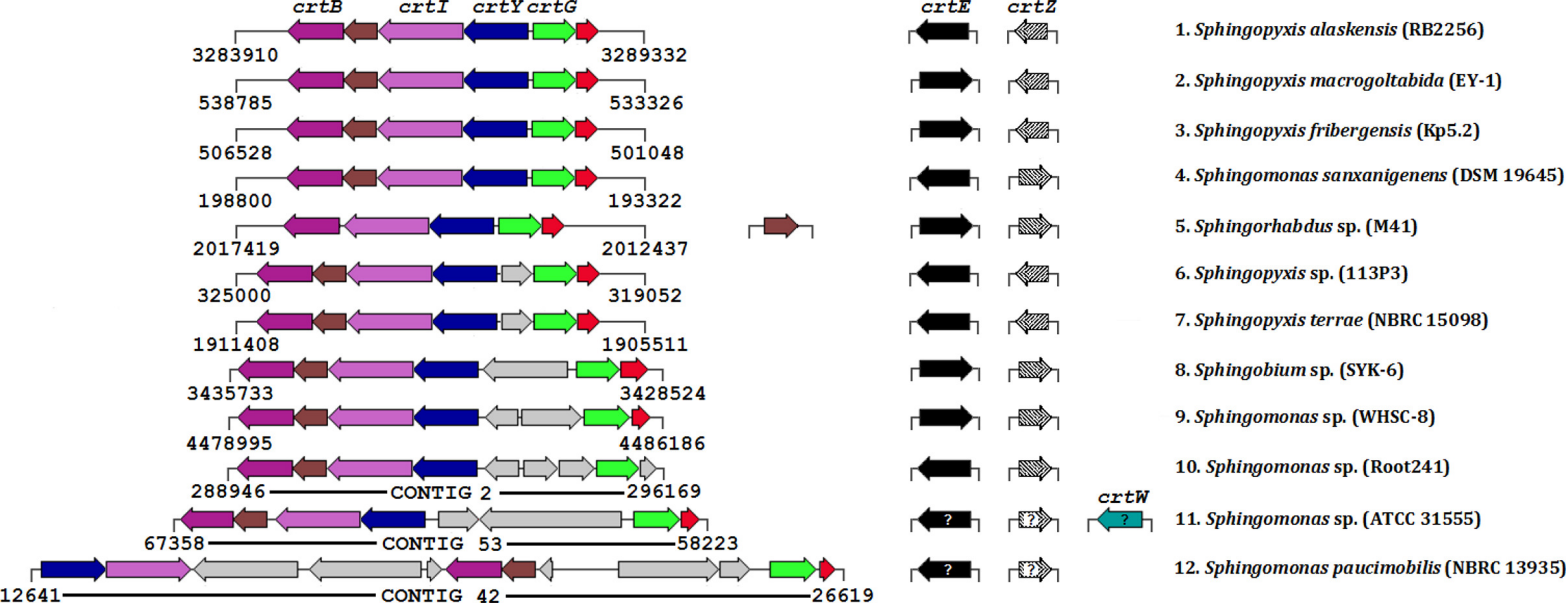
Comparison of *crt* ORFs/loci in 12 strains of *Sphingomonadales*. Serial numbers of the bacteria are the same as in Table 1. The *crt* ORF that each colour-coded or shaded arrow represents is indicated on the top. The brown and red arrows represent ORFs encoding the putative LOG and DUF2141 proteins, respectively. The grey arrows represent the *separating* ORFs that are unlikely to be involved in carotenoid biosynthesis. Numbers at the beginning and end of gene clusters indicate the coordinates within each genome or contig. In the case of *crtE* and *crtZ*, arrows pointing to the right indicate that they were located on the *plus* strand, while those pointing to the left indicate that they were located on the *minus* strand. The uncertainty of the location of these ORFs on the *plus* or *minus* strands in the draft genomes of strains ATCC 31555 and NBRC 13935 is denoted by a ‘?’ within the respective arrows.

A gene cluster is defined as a ‘set of functionally related genes located in close physical proximity in a genome’ [25]. Previous studies have shown that ORFs encoding proteins involved in C_40_/C_50_ carotenoid biosynthesis form a cluster in the chromosomes of some bacteria [26–29]. In *Sm*. *elodea* strain ATCC 31461, which is yellow pigmented and shown to produce nostoxanthin, a carotenoid biosynthesis (*crt*) locus containing seven ORFs was identified [21]. Only four of these ORFs were assigned functions based on genetic analyses and were designated *crtB*, *crtI*, *crtY* and *crtG* [21]. blastp analyses of the genomes of 41 strains (Table 1) of *Sphingomonadales* indicated that the *crt* ORFs were adjacent to each other only in five of them (Fig. 1, serial numbers 1–5). The *crt* loci within these genomes contained a set of 5–6 ORFs, wherein the *crtG* ORF appeared to be located on the ‘*opposite’* strand (Fig. 1).

Although the arrangement of the *crt* ORFs was similar in six other genomes (Fig. 1, serial numbers 6–11), *crtG* in these loci was separated from the *crtY*, *crtI* and *crtB* ORFs by the insertion of 1–3 ORFs. The draft genome of the yellow-pigmented *Sphingomonas paucimobilis* NBRC 13935 appeared to contain an atypical *crt* locus, wherein *crtG, crtB* and *crtI* were set apart from each other (Fig. 1). Furthermore, *crtY* and *crtI* were translocated upstream of *crtB*, and were present on the same strand as *crtG* in this genome (Fig. 1). All 12 genomes compared in Fig. 1 contained *crtE* and *crtZ* [encoding a putative geranylgeranyl pyrophosphate (GGPP) synthase and a β-carotene hydroxylase (CrtZ), respectively]. In each strain, these two ORFs were present in different regions of the respective chromosomes and were not part of the *crt* locus. The genome of *Sphingomonas* sp. ATCC 31555 contained *crtW* (encoding a putative β-carotene ketolase) that was also not part of the *crt* locus (Fig. 1).

The *separating* ORFs in most of the *crt* loci shown in Fig. 1 were unrelated to each other, and their roles in carotenoid biosynthesis remain to be investigated. *Sm. paucimobilis* NBRC 13935 appeared to be a prototype for the separation of the *crt* ORFs in species and strains of *Sphingomonadales* that were capable of pigment biosynthesis, because two distinct patterns of separation were observed among 23 other genomes. In 21 genomes, *crtY*, *crtI* and *crtB* occurred together (all oriented in the same direction), and were set apart from *crtG* (Fig. S1, available in the online Supplementary Material, serial numbers 14–34). In two genomes, *crtY*, *crtI* and *crtG* occurred together, and were set apart from *crtB* (Fig. S1, serial numbers 35 and 36). Notably, only seven genomes (serial numbers 13, 21, 24, 25, 30, 35 and 36) contained *crtW*. Based on these patterns, it appears that in vast majority of species and strains, *crtY*, *crtI* and *crtB* occur together, and that each of the other four ORFs (*crtE*, *crtG*, *crtZ* and *crtW*) is located elsewhere. It also appears that in at least 50 % of the genomes, *crtB* and *crtG* are oriented opposite to each other, irrespective of whether they are located close to each other or are set apart. Although *crtY*, *crtI*, *crtB* and *crtG* are located together in a single locus in a few species and strains (Fig. 1), it appears that their clustering is not required for pigmentation (e.g. in the orange- pigmented *Sphingomonas taxi* ATCC 55669, *crtB* is set apart, and in the yellow-pigmented *Sphingobium japonicum* UT26S, *crtB* and *crtG* are set apart; Fig. S1, serial numbers 35 and 37). Genome comparisons revealed that at least five ORFs (*crtE, crtB, crtI*, *crtY* and *crtZ*) are commonly found in bacteria producing carotenoid pigments. Furthermore, *Sphingomonas* sp. MM-1, *Sphingobium baderi* DE-13, *Sphingomonas wittichii* RW1 and *Sphingopyxis granuli* TFA were found to be lacking one or more of these five *crt* ORFs (Table S1), and some of them have been reported to be non-pigmented (Table 1).

### Co-occurrence of *crtY* and *crtI* is an evolutionarily conserved feature

Operonic organization and overlapping of ORFs are the hallmark of prokaryotic genomes, and these features have functional consequences [30, 31]. When the sequences of carotenoid biosynthesis genes from the purple non-sulfur photosynthetic bacterium *Rhodobacter capsulatus* were described, it was noted that the start codon (ATG) of *crtB* overlapped with the stop codon (TGA) of *crtI* [32]. Similar physical linkages of the *crt* ORFs have been observed in *Pantoea ananatis* (previously *Erwinia uredovora*) [26], *Paracoccus* sp. strain N81106 (previously *Agrobacterium aurantiacum*) [33], *Bradyrhizobium* sp. ORS278 [34] and *Sphingobium yanoikuyae* XLDN2-5 [22]. Overlapping of *crt* ORFs is likely to facilitate coordinated gene expression, and it has been shown that the preceding ORFs (e.g. *crtY* and *crtI*) contain the ribosome binding site (the Shine–Dalgarno sequence) for the subsequent ORFs (e.g. *crtI* and *crtB*, respectively) for translational coupling [33]. Genome comparisons showed that *crtY* and *crtI* occur together and have the same orientation in 38 strains of *Sphingomonadales* (Figs 1 and S1). In 28 of these strains, the reading frames of *crtY* and *crtI* overlapped by 1–8 bp, with 4 bp being the most common overlap (Table S2). In 10 strains where *crtY* and *crtI* occur together without an overlap of the ORFs, their separation range was only 6–25 bp (Table S2). Thus, it appears that the co-occurrence of *crtY* and *crtI* is an evolutionarily conserved feature in carotenogenic bacteria.

The N-terminal regions of bacterial CrtY (lycopene cyclase) and CrtI (phytoene desaturase/dehydrogenase) contain characteristic flavin adenine dinucleotide (FAD)-binding motifs, and the respective enzymes from *P. ananatis* have been shown to require FAD for their catalytic activity [35–37]. Sequence analyses showed that the N-terminal regions of most of the CrtY and CrtI orthologues from *Sphingomonadales* contain ‘GxGxxG(x)_19_E’ and ‘GxGxxG(x)_17_E’ motifs, respectively (data not shown). Although *crtI* in *P. ananatis* is located on a plasmid, the identity between the protein it encodes (GenBank accession no. ADD79328) and the CrtI orthologues of *Sphingomonadales* was high (50–59 %). In this context, the structural and functional similarities between different CrtI homologues need to be elucidated.

### *crt* loci in many strains contain an ORF encoding a putative homologue of LONELY GUY (LOG: cytokinin phosphoribohydrolase)

In bacteria that contain *crt* loci, *crtB* usually occurs immediately downstream of *crtI*, and in some cases their ORFs overlap [26–28, 33, 34]. Such occurrence appears to be rare among *Sphingomonadales*, because it was found only in three strains: *Sphingorhabdus* sp. M41, *Sphingomonas astaxanthinifaciens* DSM 22298 and *Croceicoccus naphthovorans* PQ-2 (Figs 1 and S1). Surprisingly, in the genomes of 24 strains of *Sphingomonadales*, an unusual ORF was present between *crtI* and *crtB* (Figs 1 and S1). This ORF encodes a putative protein that appeared to be a homologue of LOG involved in the direct pathway of biosynthesis of cytokinins in plants and bacteria [38–40]. Furthermore, most strains of *Sphingomonadales* had a homologue of *miaA* [which encodes a putative tRNA delta(2)-isopentenylpyrophosphate transferase (dimethylallyl diphosphate:tRNA dimethylallyltransferase)] (data not shown). In bacteria, MiaA prenylates tRNA and generates tRNA i^6^A_37_, which is a precursor for cytokinin biosynthesis [40, 41].

**Fig. 2.**
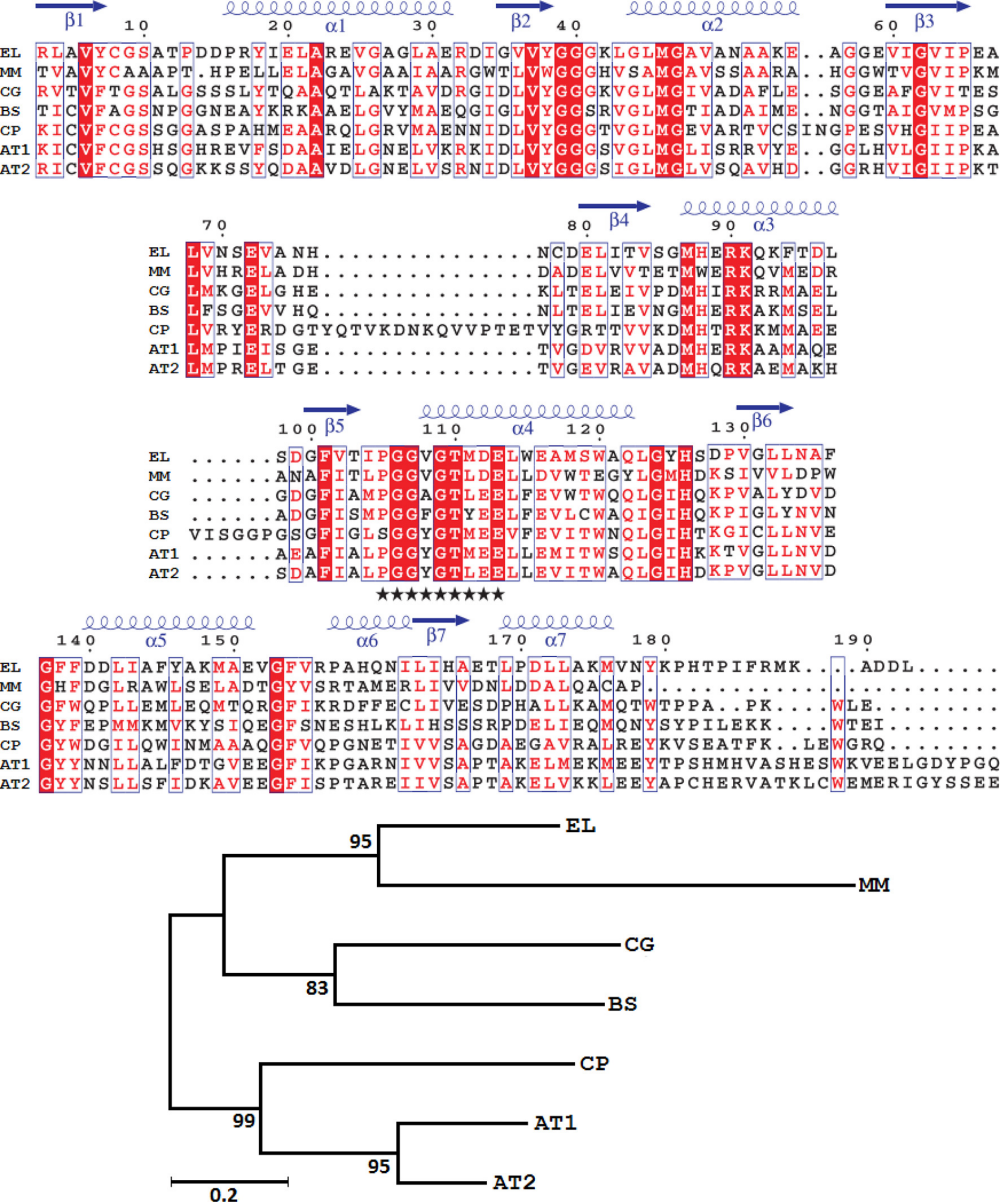
Comparison of LOG homologues. Sequences of the putative LOG (ELI_09890, 193 aa) of *Erythrobacter litoralis* (EL) and LOG of *Mycobacterium marinum* (MM: PDB code 3SBX), *Corynebacterium glutamicum* (CG: 5ITS), *Bacillus subtilis* (BS: 1T35), *Claviceps purpurea* (CP: 5AJT) and *Arabidopsis thaliana* (AT1 : 2A33, AT2 : 1YDH) were aligned. The secondary structure of ELI_09890 was inferred from the homology model constructed using 3SBX as the template (data not shown). Coils (α1–α7) and arrows (β1–β7) represent the predicted helices and strands, respectively, within ELI_09890. Residues identical in all seven homologues are shaded red, and sites with ≥70 % similarity are in red font. Blocks of conserved sites are boxed. Asterisks denote the conserved PGGxGTxxE motif. The numbers above the sequence indicate the aa positions within ELI_09890. The tree (derived using the maximum-likelihood method in mega 7.0 with the JTT substitution model) shows the phylogenetic relationship among the seven LOG homologues. All positions with less than 95 % site coverage were eliminated, and the final dataset contained 175 positions. The percentage of replicate trees in which the associated taxa clustered together in the bootstrap test (500 replicates) are shown next to the branches. The tree is drawn to scale. Bar, the number of amino acid substitutions per site.

A search of the PDB using the putative LOG of *Erythrobacter litoralis* HTCC2594 (ELI_09890, 193 aa) showed that it was related to the predicted LOG, nucleotide-binding or molybdenum cofactor carrier proteins from other bacteria and eukaryotes. Although phylogenetic analysis showed that the LOG homologues from bacteria and eukaryotes cluster on different branches, the secondary structural features of these proteins were found to be conserved (Fig. 2). Furthermore, the characteristic PGGxGTxxE motif previously identified in LOG homologues [38, 40] was conspicuous in all of them (Fig. 2). The secondary structure comparison further supports the annotation of ELI_09890 as LOG, and a model of the 3D structure (data not shown) indicates that it may be a nucleotide-binding protein.

It appears that LOG is not essential for bacterial survival [42], and has no direct role in carotenogenesis since deletion of the ORF encoding the putative LOG in *Sm. elodea* ATCC 31461 did not affect the pigment profile [21]. However, previously it has been shown that the cytokinin 6-benzylaminopurine enhances carotenoid accumulation in radish seedlings [43], *Ricinus* cell cultures [44] and the green alga *Chlorella pyrenoidosa* [45]. Furthermore, it has been reported that the deletion of *crtR*, which encodes a MarR-type repressor of the *crt* operon, enhances carotenoid accumulation in *Corynebacterium glutamicum* ATCC 13032, and that GGPP and IPP (isopentenyl diphosphate) prevent the binding of CrtR to its target DNA [29]. In view of these observations, the role of cytokinins and their precursors in bacterial carotenogenesis remains to be investigated. While cytokinin biosynthesis and the function of LOG homologues have been reported in bacteria [40, 42, 46, 47], this is the first time such homologues have been discovered within *crt* loci.

### *crtG* is co-located with a hitherto unidentified ORF in many strains

Carotenoid 2,2′-β-hydroxylase (CrtG) was first identified in *Brevundimonas* sp. strain SD212 [28]. This is a key enzyme that can convert zeaxanthin to 2, 2′-dihydroxyzeaxanthin, and astaxanthin to 2-hydroxyastaxanthin [28]. Homologues of *crtG* appear to be present in many branches of the bacterial domain, including the cyanobacterium *Thermosynechococcus elongatus* strain BP-1 [48]. The identity between CrtG of strain SD212 (GenBank accession no. BAD99415) and the CrtG orthologues from *Sphingomonadales* was rather low (40–48 %). The most conspicuous feature of *crtG* in many strains of *Sphingomonadales* was its co-location with another ORF (Figs 1 and S1). The putative proteins encoded by this ORF in different strains varied in their lengths (data not shown). blastp analyses showed that these orthologues belong to the DUF2141 superfamily of proteins that have no known function. Interestingly, members of this superfamily are found only among bacteria and have received very little attention most likely because they are not essential for cell survival. Further analyses are required to establish the role, if any, of proteins of the DUF2141 superfamily in carotenoid biosynthesis or modification.

### *crtZ* is co-located with *crtW* in some strains

The ORF encoding β-carotene hydroxylase (also referred to as carotenoid 3,3′-β-hydroxylase), which catalyses the hydroxylation of β-carotene to zeaxanthin, was identified in *P. ananatis* and was designated *crtZ* [26]. Homologues of *crtZ* have been found in numerous pigmented members of the class *Alphaproteobacteria*, and a *crtZ* deletion mutant of *Sm. elodea* ATCC 31461 has been shown to lack 3-hydroxy carotenoids [21]. Furthermore, *crtZ* of *Sphingomonas lacus* strain PB304 has been shown to convert β-carotene to zeaxanthin in an *Escherichia coli* strain that expresses CrtE (GGPP synthase), CrtB, CrtI and CrtY homologues from *Pantoea agglomerans* [23]. Multiple sequence alignment revealed the presence of two His-rich motifs (HxxHH near the N-terminal and HxLHH near the C-terminal) among the CrtZ orthologues of 37 strains of *Sphingomonadales* (data not shown). Although *crtZ* was located away from *crtY*, *crtI*, *crtB* and *crtG* in 36 strains, it was located adjacent to *crtB*, but in the opposite orientation, in *Sm. astaxanthinifaciens* DSM 22298 (Fig. S1). A similar occurrence has been reported in the *crt* locus of the related strain PB304 [23].

**Fig. 3.**
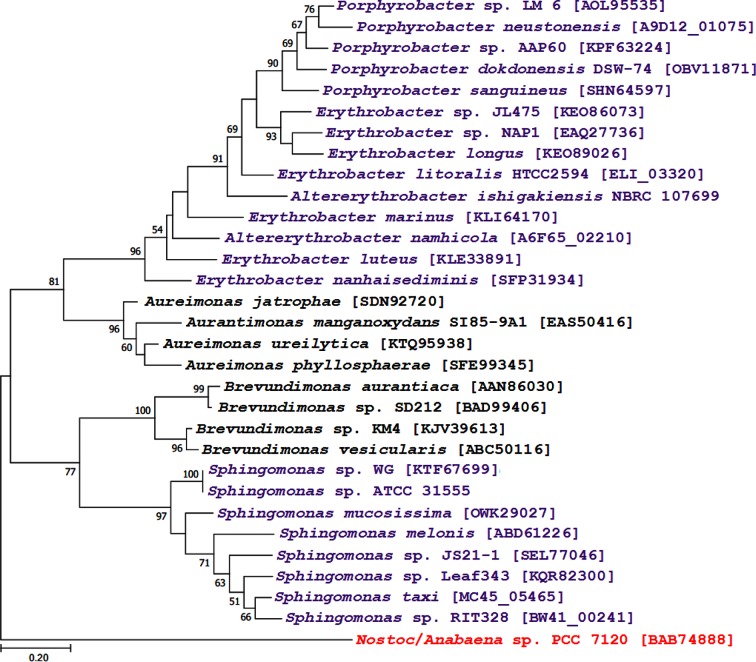
Phylogenetic tree based on CrtW homologues. The analysis involved 31 sequences and the tree was constructed using the maximum-likelihood method (with the LG+F substitution model) in mega 7.0. Bootstrap values of 1000 replicates are indicated as numbers out of 100 at the nodes (only values >50 are shown). All positions containing gaps and missing data were eliminated, and the final dataset contained 214 positions. Purple text indicates CrtW from strains of *Sphingomonadales*. Black text indicates CrtW from taxa distantly related to *Sphingomonadales*. Red text indicates the outgroup CrtW sequence. GenBank accession numbers are provided in square brackets. The tree is drawn to scale. Bar, the number of amino acid substitutions per site.

The ORF encoding β-carotene ketolase (also referred to as carotenoid 4,4′-β-oxygenase or carotenoid 4,4′-β-ketolase), which catalyses the conversion of β-carotene to canthaxanthin via echinenone, was discovered in *Haematococcus pluvialis* [49, 50]. A homologue of this gene was identified in two marine bacteria, and was designated *crtW* [33, 51]. Furthermore, *crtW* was located adjacent to *crtZ* and *crtB* in *Paracoccus* sp. strain N81106 and *Bradyrhizobium* sp. strain ORS278, respectively [33, 34]. In *Brevundimonas* sp. strain SD212 (where *crtW* was located adjacent to *crtY*, but in the opposite orientation) the presence of *crtZ*, *crtW* and *crtG* was predicted to bestow the ability to synthesize adonixanthin, astaxanthin, canthaxanthin and erythroxanthin [28]. Genomic analyses indicated that of the 37 strains of *Sphingomonadales* that contained *crtZ*, only 8 (~22 %) contained *crtW* (Figs 1 and S1). In four strains (JCM 16345, NBRC 107699, HTCC2594 and DSM 9434, which are members of *Erythrobacteraceae*), *crtW* was located immediately upstream of *crtZ* (Fig. S1). Conversely, in two strains (ATCC 55669 and RIT328, which are members of *Sphingomonas*), *crtW* was located immediately downstream of *crtZ* (Fig. S1). Interestingly, in two other strains (ATCC 31555 and DSM 22298, which are also members of *Sphingomonas*), *crtW* was located away from other *crt* ORFs (Figs 1 and S1). Three His-rich motifs (HDxxH, HxxHH and HxxHH) have been shown to be essential for β-carotene ketolase activity in *Paracoccus* sp. strain N81106 [52], and all three of them were conserved in the diverse set of CrtW (β-carotene ketolase) proteins from *Sphingomonadales* and other bacteria (data not shown).

blastp and psi-blast analyses showed that the top hits of CrtW of strains ATCC 55669 and RIT328 were from strains of the genus *Sphingomonas* and other bacteria (e.g. *Brevundimonas* spp.), but not from strains of *Erythrobacteraceae*. Similarly, the top hits of CrtW of strains JCM 16345, NBRC 107699, HTCC2594 and DSM 9434 were from strains of *Erythrobacteraceae* and other bacteria (e.g. *Aureimonas* spp.), but not from strains of the genus *Sphingomonas*. Phylogenetic analysis confirmed these results: CrtW of strains of *Sphingomonas* spp. clustered on a main branch that contained CrtW of *Brevundimonas* spp., whereas CrtW of strains of *Erythrobacteraceae* clustered on yet another main branch that contained CrtW of *Aureimonas* spp. (Fig. 3). Further analyses are required to establish whether these outcomes, and the rarity of CrtW among strains of *Sphingomonadales*, are due to preferential acquisition (through horizontal gene transfer) or differential gene loss. Similar interpretations have been made previously based on phylogenetic analysis of carotenoid hydroxylases and ketolases from various bacteria [53]. Despite their importance in carotenoid biosynthesis, many aspects of the catalytic activity of CrtZ, CrtW and CrtG remain unknown because no crystal structures are available for these proteins, and attempts to purify them for *in vitro* enzymatic characterisation were reportedly futile [54].

### *crtX* appears to be very rare among strains of *Sphingomonadales*

An ORF encoding a zeaxanthin glycosyltransferase was identified in the *crt* locus of *P. ananatis* and was designated *crtX* [26]. Homologues of this ORF have been characterised only in a few bacteria, including *P. agglomerans* [55], *Xanthobacter autotrophicus* [56] and *Paracoccus haeundaensis* [57]. An ORF encoding a putative carotenoid glycosyltransferase (CrtX, 407 aa) was identified immediately downstream of *crtW* in *Sm. lacus* PB304 [23]. A homologue of this ORF was found in *Sm. astaxanthinifaciens* DSM 22298 (68 % identity across 392 aa). The arrangement of *crtW* and *crtX* was similar in strains PB304 and DSM 22298, which were isolated from aquatic sources in Japan and Korea, respectively [58, 59]. Furthermore, the closest homologues of CrtX of these strains were found in *Aureimonas* and *Aurantimonas* (data not shown). The function of *crtX* in strains PB304 and DSM 22298, which have been reported to produce astaxanthin dideoxyglycosides [23, 60], is unknown.

### *crtE* occurs even in non-carotenogenic strains of *Sphingomonadales*

Although the condensation of two molecules of GGPP by phytoene synthase (CrtB) to produce phytoene, which is a C_40_ hydrocarbon and a colourless carotene, is the first committed step in carotenoid biosynthesis, there is reason to believe that the pathway in many organisms actually starts with the production of GGPP. This belief is supported by the fact that *crtE*, which encodes GGPP synthase, is part of the *crt* locus in some bacteria [28]. However, *crtE* was not a part of the *crt* locus either in *Sm. elodea* ATCC 31461 or in *Sm. lacus* PB304 [21, 23]. The same appeared to be true in the current comparisons, because *crtE* never co-occurred with any other *crt* ORF (Figs 1 and S1). Notably, most strains appeared to contain a single homologue of *crtE*, and strains that were non-carotenogenic also contained *crtE*. Furthermore, the CrtE orthologues of most strains of *Sphingomonadales* contained ~300 aa (Table S1), and multiple sequence alignment revealed the presence of five motifs (GKxxR, DDxxxxD, GQxxD, KT and DDxxx; data not shown) that have been shown to be conserved in bacterial, archaeal, yeast, plant and human GGPP synthases [61]. Isoprenoids and isoprenyl diphosphates are substrates for the synthesis of a variety of biologically important compounds, and *E. coli* reportedly contains three isoprenyl diphosphate synthases, but lacks a GGPP synthase [62]. In this context, the function of *crtE* in non-carotenogenic strains of *Sphingomonadales* requires further characterisation.

### PGCs are not pervasive among strains of *Sphingomonadales*

As noted previously, only a few Bchl *a*-containing species/strains of *Sphingomonadales* have been described, and PGCs were identified in the genomes of only four strains [14, 15, 24]. Comparative analysis revealed that similar PGCs also occur in the genomes of *Sphingomonas sanxanigenens* DSM 19645, *Sphingomonas hengshuiensis* WHSC-8, *Sm. astaxanthinifaciens* DSM 22298 and *Altererythrobacter ishigakiensis* NBRC 107699 (Table 1). However, the latter strain was reported to lack Bchl *a* [63], and no information is available about the presence or absence of Bchl *a* in the descriptions of the type strains DSM 22298, DSM 19645 and WHSC-8 [57, 64, 65]. Nevertheless, it is evident from Table 1 that PGCs are generally found in strains that are coloured, and strains that are non-carotenogenic due to the absence of one or more *crt* ORFs lack PGCs. Whether β-carotene and its derivates have any role in anoxygenic phototrophy in *Sphingomonadales* remains to be investigated.

### Conclusions

**Fig. 4.**
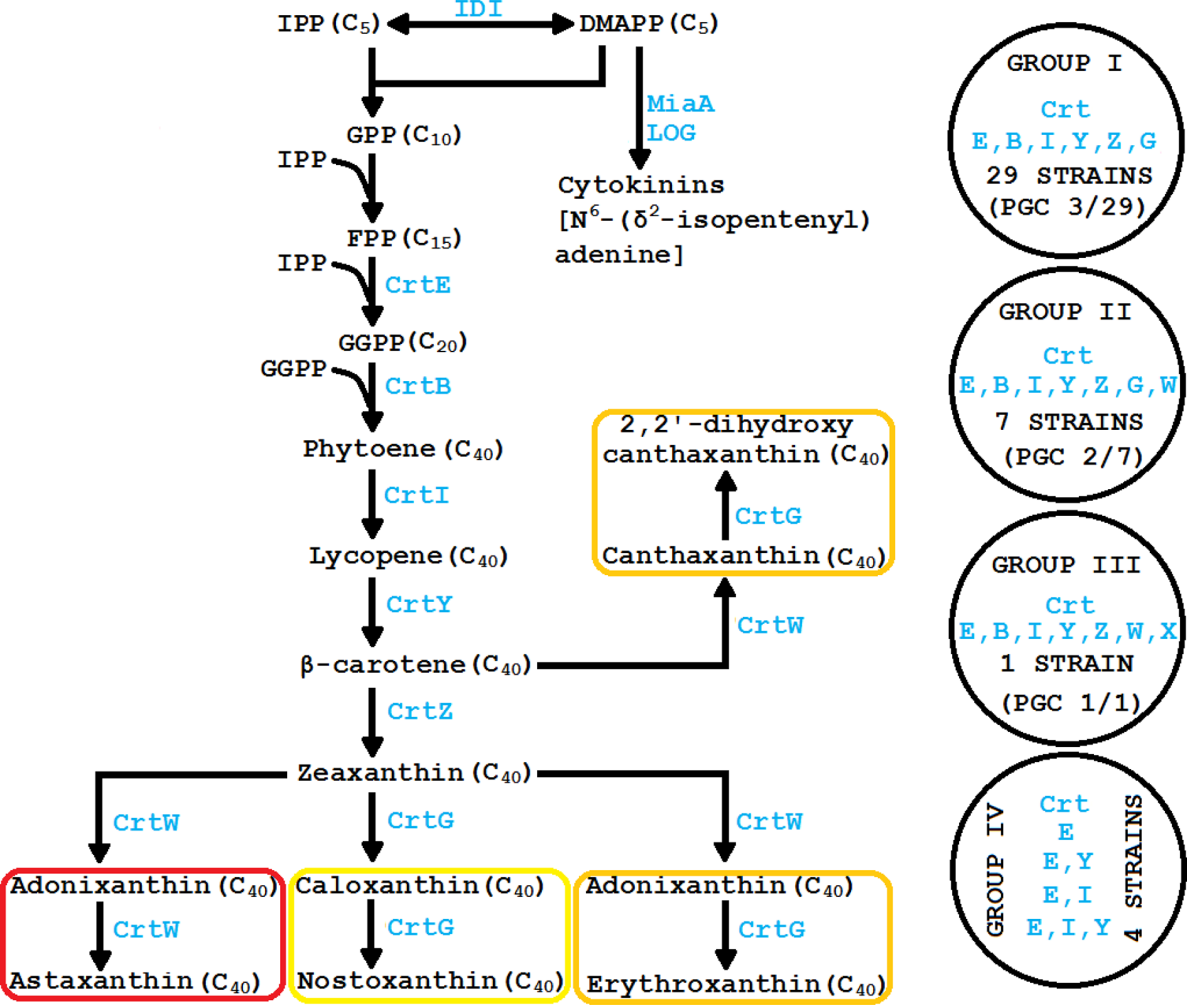
Presumptive pathways of carotenoid biosynthesis in strains of *Sphingomonadales*. Shorthand notations of enzymes are indicated in blue (e.g. IDI – isopentenyl diphosphate isomerase). Zeaxanthin (whose biosynthesis is enabled by the presence of CrtE, CrtB, CrtI, CrtY and CrtZ) appears to be the branchpoint for three different pathways that produce astaxanthin (using CrtW), nostaxanthin (using CrtG) and erythroxanthin (using CrtW and CrtG). Additionally, β-carotene is the branchpoint for the pathway that produces 2,2′-dihydroxycanthaxanthin (using CrtW and CrtG). The precursor for cytokinin production (using MiaA and LOG) and carotenoid biosynthesis is DMAPP. Circles on the right represent the grouping of bacterial strains based on their *crt* genotype as shown in Table 1.

This study sought to understand the genetic basis for the vibrant colours of members of the *Sphingomonadales* through comparative genomic analysis. It was found that the *crt* ORFs were scattered across the chromosome in many strains, indicating that clustering of *crt* ORFs is not necessary for the coloured phenotype. Based on the current analyses, strains of *Sphingomonadales* could broadly be categorised into those that are coloured (with subcategories of yellow, orange and red) and colourless. Yellow coloured strains most likely accumulate nostoxanthin, and contain six ORFs (group I: *crtE*, *crtB*, *crtI*, *crtY*, *crtZ*, *crtG*; Table 1, Fig. 4). However, orange coloured strains (group II: *crtE*, *crtB*, *crtI*, *crtY*, *crtZ*, *crtG*, *crtW*; Table 1, Fig. 4) could contain some of the carotenoids predicted to be produced by *Brevundimonas* sp. strain SD212 [28], in addition to echinenone, zeaxanthin, caloxanthin and/or nostoxanthin. Red coloured strains may contain six (*crtE*, *crtB*, *crtI*, *crtY*, *crtZ*, *crtW*) or seven (*crtE*, *crtB*, *crtI*, *crtY*, *crtZ*, *crtW*, *crt*X) ORFs (group III; Table 1, Fig. 4) that enable the production of astaxanthin and its derivatives. Non-pigmented strains may contain a smaller subset of *crt* ORFs, and are thus unable to produce any carotenoids (group IV; Table 1, Fig. 4). The four groups appear to transcend phylogenetic relationships within the order *Sphingomonadales*.

It is important to note that the relative quantities of the different carotenoids (and their isomers) could determine the type and intensity of colour. This notion is supported by the observation that cells containing lower levels of lycopene are pink, whereas those accumulating higher levels can be red [21, 33, 37, 66–69]. Similarly, cells containing low/moderate levels of β-carotene are yellow, whereas those accumulating higher levels can be orange [23, 26, 33, 35, 36, 67, 70–72]. Zeaxanthin producing cells are no exception in this context; they can be yellow, orange or yellow–orange [23, 26, 65, 73–76]. Furthermore, the absorption spectra of β-carotene, zeaxanthin, caloxanthin and nostoxanthin are apparently similar. Thus, the *crt* genotype identified in strains of *Sphingomonadales* will have to be reconciled with the ‘actual’ colour phenotype and vice-versa. Occasionally, some strains of this order could contain the minimal *crt* genotype to be coloured, but appear colourless (e.g. *Sm. sanxanigenens* DSM 19645 and *Sphingorhabdus* sp. M41). Such strains could accumulate GGPP or phytoene, which are colourless compounds, due to dysfunctional *crtB* or *crtI*, respectively. They could also be colourless due to repression of *crtB* and/or *crtI* by as yet unknown mechanisms.

## Data bibliography

1. Lauro FM *et al.* GenBank accession no. CP000356 (2006).
2. Ohtsubo Y *et al.* GenBank accession no. CP012700 (2015).
3. Oelschlagel M *et al.* GenBank accession no. CP009122 (2014).
4. Ma T *et al.* GenBank accession no. CP006644 (2013).
5. Jeong H *et al.* GenBank accession no. CP014545 (2015).
6. Ohtsubo Y *et al.* GenBank accession no. CP009452 (2015).
7. Ohtsubo Y *et al.* GenBank accession no. CP013342 (2015).
8. Masai E *et al.* NCBI RefSeq accession no. NC_015976 (2011).
9. Wei S *et al.* GenBank accession no. CP010836 (2015).
10. Bai Y *et al.* NCBI RefSeq accession no. NZ_LMIV01000000 (2015).
11. Wang X *et al.* NCBI RefSeq accession no. NZ_ALBQ00000000 (2012).
12. Hosoyama A *et al.* NCBI RefSeq accession no. NZ_BBJS01000000 (2014).
13. Kyrpides N *et al.* NCBI RefSeq accession no. NZ_JONN00000000 (2014).
14. Lucas S *et al.* GenBank accession no. CP002798 (2011).
15. Tabata M *et al.* GenBank accession no. CP005188 (2013).
16. Lee C-M *et al.* GenBank accession no. CP015986 (2016).
17. Yan X. GenBank accession no. CP010954 (2015).
18. Copeland A *et al.* GenBank accession no. CP000248 (2006).
19. Zheng Q *et al.* GenBank accession no. CP011344 (2015).
20. Cheng H *et al.* GenBank accession no. CP016591 (2016).
21. Cheng H *et al.* GenBank accession no. CP016545 (2016).
22. Li Z *et al.* GenBank accession no. CP012669 (2015).
23. Wu YH *et al.* GenBank accession no. CP011452 (2015).
24. Wu YH *et al.* GenBank accession no. CP015963 (2016).
25. Giovannoni SJ *et al.* GenBank accession no. CP000157 (2005).
26. Zhuang L *et al.* GenBank accession no. CP011310 (2015).
27. Earl A *et al.* NCBI RefSeq accession no. NZ_AGZU00000000 (2012).
28. Petrillo M. GenBank accession no. FR856862 (2011).
29. Choi DH *et al.* GenBank accession no. CP009291 (2014).
30. Shi XL *et al.* GenBank accession no. CP016033 (2016).
31. Zhu X *et al.* GenBank accession no. CP015521 (2016).
32. Wang H, Zhu P. NCBI RefSeq NZ_LQCK00000000 (2015).
33. Kim KM. GenBank accession no. CP011805 (2015).
34. Zeng Y, Huang Y. GenBank accession no. CP011770 (2015).
35. Zhou Y *et al.* GenBank accession no. CP009571 (2014).
36. Gan HY *et al.* NCBI RefSeq accession no. NZ_JFYV01000000 (2014).
37. Nagata Y *et al.* NCBI RefSeq accession no. NC_014006 (2008).
38. Tabata M *et al.* GenBank accession no. CP004036 (2013).
39. Cheng M *et al.* GenBank accession no. CP013264 (2015).
40. Copeland A *et al.* GenBank accession no. CP000699 (2007).
41. Garcia-Romero I *et al.* GenBank accession no. CP012199 (2015).

## Supplementary Data

946 Supplementary File 1
