# Supplementary material for: Genomewide characterisation of the genetic diversity of carotenogenesis in bacteria of the order Sphingomonadales
Source: Microb Genom. 2018 Apr 5;4(4):e000172. doi: 10.1099/mgen.0.000172 (PMC5989583; doi:10.1099/mgen.0.000172)
Supplement: Supplementary File 1 [file mgen-4-172-s001.pdf]

Table S1. Locus tags of putative proteins that may have a direct/indirect role in carotenoid biosynthesis in *Sphingomonadales*

	**CrtE**	**CrtB**	**LOG**	**CrtI**	**CrtY**	**CrtG**	**DUF2141***	**CrtZ**	**CrtW**
1	Sala_2194<br>306 aa	Sala_3132<br>327 aa	Sala_3133<br>193 aa	Sala_3134<br>492 aa	Sala_3135<br>385 aa	Sala_3136<br>253 aa	Sala_3137<br>119 aa	Sala_2128<br>172 aa	None
2	AN936_01555<br>305 aa	AN936_02675<br>316 aa	AN936_02670<br>193 aa	AN936_02665<br>491 aa	AN936_02660<br>385 aa	AN936_02655<br>256 aa	AN936_02650<br>119 aa	AN936_16185<br>172 aa	None
3	SKP52_03550<br>306 aa	SKP52_02265<br>319 aa	SKP52_02260<br>193 aa	SKP52_02255<br>490 aa	SKP52_02250<br>385 aa	SKP52_02245<br>255 aa	SKP52_02240<br>119 aa	SKP52_17370<br>172 aa	None
4	NX02_22505<br>293 aa	NX02_00980<br>312 aa	NX02_00975<br>192 aa	NX02_00970<br>494 aa	NX02_00965<br>393 aa	NX02_00960<br>265 aa	NX02_00955<br>119 aa	NX02_21750<br>172 aa	None
5	AZE99_02430<br>300 aa	AZE99_09605<br>311 aa	AZE99_15750<br>189 aa	AZE99_09600<br>503 aa	AZE99_09595<br>388 aa	AZE99_09590<br>254 aa	AZE99_09585<br>120 aa	AZE99_11975<br>171 aa	None
6	LH20_15970<br>307 aa	LH20_01385<br>319 aa	LH20_01380<br>193 aa	LH20_01375<br>491 aa	LH20_01370<br>386 aa	LH20_01360<br>252 aa	LH20_01355<br>119 aa	LH20_13675<br>172 aa	None
7	AOA14_02810<br>299 aa	AOA14_09220<br>323 aa	AOA14_09215<br>193 aa	AOA14_09210<br>490 aa	AOA14_09205<br>385 aa	AOA14_09195<br>262 aa	AOA14_09190<br>119 aa	AOA14_05275<br>172 aa	None
8	SLG_30760<br>300 aa	SLG_31750<br>321 aa	SLG_31740<br>196 aa	SLG_31730<br>492 aa	SLG_31720<br>387 aa	SLG_31700<br>249 aa	SLG_31690<br>120 aa	SLG_38860<br>174 aa	None
9	TS85_16025<br>299 aa	TS85_20435<br>311 aa	TS85_20440<br>193 aa	TS85_20445<br>491 aa	TS85_20450<br>393 aa	TS85_20465<br>262 aa	TS85_20470<br>119 aa	TS85_19965<br>166 aa	None
10	ASE13_12915<br>302 aa	ASE13_12025<br>318 aa	ASE13_12030<br>193 aa	ASE13_12035<br>492 aa	ASE13_12040<br>386 aa	ASE13_12060<br>249 aa	None	ASE13_11100<br>166 aa	None
11	Sphi55_0001<br>302 aa	Sphi55_0002<br>307 aa	Sphi55_0003<br>193 aa	Sphi55_0004<br>492 aa	Sphi55_0005<br>385 aa	Sphi55_0007<br>265 aa	Sphi55_0008<br>119 aa	Sphi55_0006<br>166 aa	Sphi55_0009<br>261 aa
12	SP6_03_00020<br>299 aa	SP6_42_00220<br>316 aa	SP6_42_00230<br>193 aa	SP6_42_00180<br>491 aa	SP6_42_00170<br>390 aa	SP6_42_00290<br>271 aa	SP6_42_00300<br>119 aa	SP6_29_00510<br>179 aa	None
13	Sasta_0001<br>301 aa	Sasta_0002<br>312 aa	Sasta_0003<br>193 aa	Sasta_0004<br>487 aa	Sasta_0005<br>380 aa	None	Sasta_0008<br>120 aa	Sasta_0006<br>176 aa	Sasta_0009<br>258 aa
14	Sphch_1308<br>302 aa	Sphch_1941<br>317 aa	Sphch_1942<br>193 aa	Sphch_1943<br>493 aa	Sphch_1944<br>383 aa	Sphch_2837<br>241 aa	Sphch_2838<br>119 aa	Sphch_2465<br>167 aa	None
15	K663_05560<br>302 aa	K663_01410<br>319 aa	K663_01405<br>193 aa	K663_01400<br>493 aa	K663_01395<br>383 aa	K663_13655<br>242 aa	K663_13650<br>119 aa	K663_01820<br>170 aa	None

16	EP837_00635 302 aa	EP837_01648 319 aa	EP837_01647 193 aa	EP837_01646 493 aa	EP837_01645 383 aa	EP837_01002 242 aa	EP837_01001 119 aa	EP837_01682 170 aa	None
17	TZ53_03080 306 aa	TZ53_10675 317 aa	TZ53_10670 193 aa	TZ53_10665 493 aa	TZ53_10660 386 aa	TZ53_01450 237 aa	TZ53_01445 119 aa	TZ53_12060 169 aa	None
18	Saro_2253 303 aa	Saro_1814 323 aa	Saro_1815 193 aa	Saro_1816 492 aa	Saro_1817 398 aa	Saro_0236 236 aa	Saro_0235 121 aa	Saro_1168 172 aa	None
19	WG74_10145 302 aa	WG74_04285 324 aa	WG74_04290 195 aa	WG74_04295 500 aa	WG74_04300 403 aa	WG74_03350 240 aa	WG74_03345 122 aa	WG74_11370 172 aa	None
20	A6F68_02109 304 aa	A6F68_02787 341 aa	A6F68_02786 193 aa	A6F68_02785 497 aa	A6F68_02784 393 aa	A6F68_00557 237 aa	A6F68_00556 121 aa	A6F68_01016 172 aa	None
21	A6F65_01603 303 aa	A6F65_00929 344 aa	A6F65_00930 201 aa	A6F65_00931 508 aa	A6F65_00932 402 aa	A6F65_00501 239 aa	A6F65_00500 122 aa	A6F65_02209 166 aa	A6F65_02210 262 aa
22	AMC99_01057 301 aa	AMC99_00377 341 aa	AMC99_00378 193 aa	AMC99_00379 523 aa	AMC99_00380 394 aa	AMC99_02463 244 aa	AMC99_02464 121 aa	AMC99_01700 173 aa	None
23	WYH_00402 301 aa	WYH_03017 341 aa	WYH_03018 193 aa	WYH_03019 501 aa	WYH_03020 393 aa	WYH_02232 238 aa	WYH_02233 121 aa	WYH_00011 172 aa	None
24	Altish_0001 301 aa	Altish_0002 315 aa	Altish_0003 193 aa	Altish_0004 520 aa	Altish_0005 402 aa	Altish_0007 240 aa	Altish_0008 121 aa	Altish_0006 166 aa	Altish_0009 255 aa
25	ELI_05575 302 aa	ELI_09895 341 aa	ELI_09890 193 aa	ELI_09885 501 aa	ELI_09880 400 aa	ELI_12610 240 aa	ELI_12615 121 aa	ELI_03325 166 aa	ELI_03320 255 aa
26	CP97_10495 301 aa	CP97_13035 348 aa	CP97_13030 193 aa	CP97_13025 498 aa	CP97_13020 398 aa	CP97_01785 241 aa	CP97_01790 121 aa	CP97_11790 172 aa	None
27	HMPREF9718_03593 301 aa	HMPREF9718_02587 329 aa	HMPREF9718_02588 193 aa	HMPREF9718_02589 493 aa	HMPREF9718_02590 382 aa	HMPREF9718_03515 246 aa	HMPREF9718_03516 119 aa	HMPREF9718_02777 172 aa	None
28	PP1Y_AT24739 301 aa	PP1Y_AT8258 317 aa	PP1Y_AT8268 193 aa	PP1Y_AT8278 492 aa	PP1Y_AT8289 408 aa	PP1Y_AT36759 240 aa	PP1Y_AT36765 120 aa	PP1Y_AT11105 172 aa	None
29	JI59_00550 301 aa	JI59_14775 317 aa	JI59_14780 193 aa	JI59_14790 492 aa	JI59_14795 408 aa	JI59_11990 240 aa	JI59_11985 120 aa	JI59_06385 172 aa	None
30	A9D12_11730 303 aa	A9D12_07620 321 aa	A9D12_07625 193 aa	A9D12_07635 520 aa	A9D12_07640 425 aa	A9D12_05235 257 aa	A9D12_05230 121 aa	A9D12_01070 179 aa	A9D12_01075 267 aa
31	A7E77_04905 300 aa	A7E77_09430 314 aa	A7E77_09425 193 aa	A7E77_09415 491 aa	A7E77_09410 388 aa	A7E77_06785 270 aa	A7E77_06790 119 aa	A7E77_07315 171 aa	None
32	BJP26_00185 300 aa	BJP26_05345 314 aa	BJP26_05340 193 aa	BJP26_05325 491 aa	BJP26_05320 388 aa	BJP26_02355 270 aa	BJP26_02360 119 aa	BJP26_02830 171 aa	None

33	AM2010_812<br>302 aa	AM2010_155<br>346 aa	AM2010_156<br>193 aa	AM2010_158<br>520 aa	AM2010_159<br>393 aa	AM2010_2362<br>240 aa	AM2010_1236<br>120 aa	AM2010_1583<br>172 aa	None
34	AB433_06870<br>312 aa	AB433_09240<br>328 aa	AB433_12870<br>189 aa	AB433_09235<br>496 aa	AB433_09230<br>398 aa	AB433_15915<br>250 aa	AB433_15920<br>119 aa	AB433_00865<br>174 aa	None
35	MC45_16265<br>296 aa	MC45_14215<br>316 aa	MC45_14210<br>193 aa	MC45_14760<br>491 aa	MC45_14765<br>388 aa	MC45_14775<br>274 aa	MC45_14780<br>119 aa	MC45_05470<br>155 aa	MC45_05465<br>238 aa
36	BW41_00905<br>296 aa	BW41_00781<br>315 aa	BW41_00782<br>193 aa	BW41_03520<br>491 aa	BW41_03521<br>388 aa	BW41_03523<br>272 aa	BW41_03524<br>119 aa	BW41_00240<br>155 aa	BW41_00241<br>236 aa
37	SJA_C1-33430<br>305 aa	SJA_C1-01890<br>317 aa	SJA_C1-01590<br>193 aa	SJA_C1-01600<br>493 aa	SJA_C1-01610<br>386 aa	SJA_C1-28960<br>241 aa	SJA_C1-28950<br>119 aa	SJA_C1-06860<br>169 aa	None
38	G432_12610<br>305 aa	None	G432_07845<br>193 aa	G432_07840<br>492 aa	G432_07835<br>394 aa	None	None	None	None
39	ATN00_08710<br>303 aa	None	ATN00_06065<br>193 aa	ATN00_06060<br>493 aa	None	None	None	None	None
40	Swit_0022<br>297 aa	None	Swit_3199<br>196 aa	None	Swit_3200<br>380 aa	None	None	None	None
41	SGRAN_0972<br>307 aa	None	SGRAN_3508<br>193 aa	None	None	None	None	None	None

Serial numbers in the first column are the same as in Tables 1 and S2.
*Number of aa is counted from the first conserved substitution (V/L/I).
Sequences for locus tags in serial numbers 11, 13, and 24 are provided after Fig. S1.

Table S2. Comparison of the features of *crt* and *log* ORFs identified in strains of *Sphingomonadales*

	Species (strain)	*crtY* Length (Start. .Stop)	*crtI* Length (Start. .Stop)	Gap/overlap between *crtY* and *crtI*	Gap/overlap between *crtI* and *log*	*log* Length (Start. .Stop)	*crtB* Length (Start. .Stop)	Gap/overlap between *log* and *crtB*	*crtG* Length (Start. .Stop)	*crtZ* Length (Start. .Stop)	*crtW* Length (Start. .Stop)
1	*Sphingopyxis* alaskensis (RB2256)	1158 **(ATG. .TGA)**	1479 **(ATG. .TAA)**	Overlap (4 bp)	Gap (35 bp)	582 **(ATG. .TGA)**	984 **(ATG. .TGA)**	Overlap (1 bp)	762 **(ATG. .TGA)**	519 **(ATG. .TAA)**	None
2	*Sphingopyxis macrogoltabida* (EY-1)	1158 **(ATG. .TGA)**	1476 **(ATG. .TAA)**	Overlap (4 bp)	Gap (98 bp)	582 **(ATG. .TAA)**	951 **(ATG. .TGA)**	Overlap (1 bp)	771 **(GTG. .TGA)**	519 **(ATG. .TAG)**	None
3	*Sphingopyxis fribergensis* (Kp5.2)	1158 **(ATG. .TGA)**	1473 **(ATG. .TGA)**	Overlap (4 bp)	Gap (116 bp)	582 **(ATG. .TAA)**	960 **(ATG. .TGA)**	Overlap (1 bp)	768 **(ATG. .TGA)**	519 **(ATG. .TAA)**	None
4	*Sphingomonas sanxanigenens* (DSM 19645)	1182 **(ATG. .TGA)**	1485 **(ATG. .TGA)**	Overlap (4 bp)	Gap (28 bp)	579 **(ATG. .TGA)**	939 **(GTG. .TGA)**	Overlap (4 bp)	798 **(GTG. .TGA)**	519 **(ATG. .TGA)**	None
5	*Sphingorhabdus* sp. (M41)	1167 **(ATG. .TGA)**	1512 **(ATG. .TGA)**	Gap (13 bp)	*log* is off-located	570 **(TTG. .TGA)**	936 **(ATG. .TAG)**	*crtB* is separated from *crtI* by 75 bp	765 **(ATG. .TGA)**	516 **(ATG. .TAG)**	None
6	*Sphingopyxis* sp. (113P3)	1161 **(ATG. .TGA)**	1476 **(ATG. .TAG)**	Overlap (4 bp)	Gap (35 bp)	582 **(ATG. .TGA)**	960 **(ATG. .TGA)**	Overlap (1 bp)	759 **(ATG. .TGA)**	519 **(ATG. .TAG)**	None
7	*Sphingopyxis terrae* (NBRC 15098)	1158 **(ATG. .TGA)**	1473 **(ATG. .TGA)**	Overlap (4 bp)	Overlap (1 bp)	582 **(ATG. .TAA)**	972 **(ATG. .TGA)**	Overlap (1 bp)	789 **(ATG. .TGA)**	519 **(ATG. .TGA)**	None
8	*Sphingobium* sp. (SYK-6)	1164 **(ATG. .TGA)**	1479 **(ATG. .TGA)**	Overlap (4 bp)	Overlap (4 bp)	591 **(ATG. .TGA)**	966 **(GTG. .TGA)**	Gap (36 bp)	750 **(ATG. .TGA)**	525 **(ATG. .TAG)**	None
9	*Sphingomonas hengshuiensis* (WHSC-8)	1182 **(ATG. .TGA)**	1476 **(ATG. .TAA)**	Overlap (4 bp)	Gap (1 bp)	582 **(TTG. .TGA)**	936 **(ATG. .TAG)**	Gap (17 bp)	789 **(GTG. .TGA)**	501 **(ATG. .TGA)**	None

10	*Sphingomonas* sp. (Root241)	1161 **(ATG..TGA)**	1479 **(ATG..TGA)**	Overlap (4 bp)	Overlap (4 bp)	582 **(ATG..TGA)**	957 **(GTG..TAA)**	Overlap (4 bp)	750 **(ATG..TAG)**	501 **(ATG..TGA)**	None
11	*Sphingomonas* sp. (ATCC 31555)	1158 **(ATG..TGA)**	1479 **(ATG..TGA)**	Overlap (1 bp)	Gap (171 bp)	582 **(ATG..TAA)**	924 **(ATG..TAA)**	Overlap (1 bp)	798 **(ATG..TGA)**	501 **(ATG..TGA)**	786 **(ATG..TGA)**
12	*Sphingomonas paucimobilis* (NBRC 13935)	1173 **(ATG..TGA)**	1476 **(GTG..TAA)**	Overlap (4 bp)	Gap contains 3 ORFs	582 **(ATG..TGA)**	951 **(GTG..TAG)**	Overlap (4 bp)	816 **(GTG..TGA)**	540 **(ATG..TAA)**	None
13	*Sphingomonas astaxanthinifaciens* (DSM 22298)	1143 **(ATG..TGA)**	1464 **(GTG..TGA)**	Overlap (4 bp)	*log* is off-located	582 **(GTG..TAG)**	939 **(GTG..TGA)**	*crtB* overlaps with *crtI* by 4 bp	None	531 **(ATG..TGA)**	777 **(ATG..TGA)**
14	*Sphingobium chlorophenolicum* (L-1)	1152 **(ATG..TGA)**	1482 **(ATG..TAG)**	Gap (13 bp)	Gap (1 bp)	582 **(ATG..TGA)**	954 **(ATG..TGA)**	Gap (363 bp)	726 **(ATG..TGA)**	504 **(ATG..TAG)**	None
15	*Sphingobium* sp. (MI1205)	1152 **(ATG..TGA)**	1482 **(ATG..TGA)**	Overlap (1 bp)	Overlap (4 bp)	582 **(GTG..TGA)**	960 **(ATG..TGA)**	Gap (40 bp)	729 **(ATG..TGA)**	513 **(ATG..TGA)**	None
16	*Sphingobium* sp. (EP60837)	1152 **(ATG..TGA)**	1482 **(ATG..TGA)**	Overlap (1 bp)	Overlap (4 bp)	582 **(TTG..TAG)**	960 **(ATG..TGA)**	Gap (37 bp)	729 **(ATG..TGA)**	513 **(ATG..TAG)**	None
17	*Sphingobium* sp. (YBL2)	1161 **(ATG..TGA)**	1482 **(ATG..TGA)**	Gap (13 bp)	Gap (12 bp)	582 **(ATG..TGA)**	954 **(ATG..TGA)**	Gap (354 bp)	714 **(ATG..TAG)**	510 **(ATG..TGA)**	None
18	*Novosphingobium aromaticivorans* (DSM 12444)	1197 **(ATG..TGA)**	1479 **(ATG..TGA)**	Overlap (4 bp)	Overlap (4 bp)	582 **(GTG..TGA)**	972 **(ATG..TGA)**	Gap (4 bp)	711 **(ATG..TGA)**	519 **(ATG..TAG)**	None
19	*Citromicrobium* sp. (JL477)	1212 **(ATG..TGA)**	1503 **(ATG..TGA)**	Overlap (8 bp)	Gap (299 bp)	588 **(ATG..TGA)**	975 **(GTG..TAG)**	Gap (32 bp)	723 **(ATG..TGA)**	519 **(ATG..TAG)**	None
20	*Altererythrobacter dongtanensis* (KCTC 22672)	1182 **(ATG..TGA)**	1494 **(TTG..TGA)**	Overlap (4 bp)	Overlap (4 bp)	582 **(GTG..TGA)**	1026 **(CTG..TGA)**	Gap (61 bp)	714 **(ATG..TGA)**	519 **(ATG..TAG)**	None

21	*Altererythrobacter namhicola* (JCM 16345)	1209 **(ATG. .TGA)**	1527 **(ATG. .TGA)**	Overlap (4 bp)	Overlap (1 bp)	606 **(ATG. .TGA)**	1035 **(ATG. .TAA)**	Gap (44 bp)	720 **(ATG. .TGA)**	501 **(ATG. .TGA)**	789 **(ATG. .TGA)**
22	*Altererythrobacter epoxidivorans* (CGMCC 1.7731)	1185 **(ATG. .TGA)**	1572 **(ATG. .TGA)**	Gap (6 bp)	Gap (8 bp)	582 **(ATG. .TAA)**	1026 **(CTG. .TGA)**	Gap (61 bp)	735 **(ATG. .TGA)**	522 **(ATG. .TAA)**	None
23	*Altererythrobacter atlanticus* (26DY36)	1182 **(ATG. .TGA)**	1506 **(ATG. .TGA)**	Overlap (4 bp)	Overlap (4 bp)	582 **(GTG. .TAA)**	1026 **(CTG. .TGA)**	Gap (43 bp)	717 **(ATG. .TGA)**	519 **(ATG. .TGA)**	None
24	*Altererythrobacter ishigakiensis* (NBRC 107699)	1209 **(ATG. .TGA)**	1563 **(ATG. .TGA)**	Overlap (4 bp)	Overlap (4 bp)	582 **(CTG. .TGA)**	948 **(GTG. .TAG)**	Overlap (4 bp)	723 **(ATG. .TGA)**	501 **(ATG. .TAA)**	768 **(ATG. .TGA)**
25	*Erythrobacter litoralis* (HTCC2594)	1203 **(ATG. .TGA)**	1506 **(ATG. .TGA)**	Overlap (4 bp)	Overlap (4 bp)	582 **(ATG. .TGA)**	1026 **(CTG. .TAG)**	Gap (56 bp)	723 **(ATG. .TAG)**	501 **(ATG. .TGA)**	768 **(ATG. .TGA)**
26	*Erythrobacter atlanticus* (s21-N3)	1197 **(ATG. .TGA)**	1497 **(ATG. .TAG)**	Overlap (4 bp)	Gap (98 bp)	582 **(ATG. .TGA)**	1047 **(GTG. .TGA)**	Gap (35 bp)	726 **(ATG. .TGA)**	519 **(ATG. .TGA)**	None
27	*Sphingobium yanoikuyae* (ATCC 51230)	1149 **(GTG. .TGA)**	1482 **(ATG. .TGA)**	Overlap (1 bp)	Gap (31 bp)	582 **(ATG. .TGA)**	990 **(ATG. .TGA)**	Gap (54 bp)	741 **(ATG. .TGA)**	519 **(ATG. .TAG)**	None
28	*Novosphingobium* sp. (PP1Y)	1227 **(ATG. .TGA)**	1479 **(ATG. .TGA)**	Overlap (4 bp)	Gap (435 bp, contains an ORF)	582 **(ATG. .TGA)**	954 **(ATG. .TAG)**	Gap (37 bp)	723 **(TTG. .TGA)**	519 **(ATG. .TGA)**	None
29	*Novosphingobium pentaromativorans* (US6-1)	1227 **(ATG. .TGA)**	1479 **(ATG. .TGA)**	Overlap (4 bp)	Gap (435 bp, contains JI59_14785)	582 **(ATG. .TGA)**	954 **(ATG. .TAG)**	Gap (37 bp)	723 **(TTG. .TGA)**	519 **(ATG. .TGA)**	None
30	*Porphyrobacter neustonensis* (DSM 9434)	1278 **(ATG. .TGA)**	1563 **(ATG. .TGA)**	Overlap (4 bp)	Gap (432 bp, contains A9D12_07630)	582 **(ATG. .TAG)**	966 **(ATG. .TGA)**	Gap (11 bp)	774 **(ATG. .TGA)**	540 **(ATG. .TAA)**	804 **(ATG. .TGA)**

31	*Sphingomonas* sp. (NIC1)	1167 **(ATG..TAG)**	1476 **(ATG..TGA)**	Gap (16 bp)	Gap (832 bp, contains A7E77_09425)	582 **(ATG..TGA)**	945 **(CTG..TAG)**	Overlap (11 bp)	813 **(ATG..TGA)**	516 **(ATG..TAA)**	None
32	*Sphingomonas melonis* (TY)	1167 **(ATG..TAG)**	1476 **(ATG..TGA)**	Gap (16 bp)	Gap (1035 bp, contains BJP26_05330 BJP26_05335)	582 **(ATG..TGA)**	945 **(CTG..TAG)**	Overlap (11 bp)	813 **(ATG..TGA)**	516 **(ATG..TAA)**	None
33	*Altererythrobacter marensis* (KCTC 22370)	1182 **(ATG..TGA)**	1563 **(ATG..TGA)**	Overlap (4 bp)	Gap (510 bp, contains AM2010_157)	582 **(ATG..TGA)**	1041 **(CTG..TAG)**	Gap (61 bp)	723 **(ATG..TAG)**	519 **(ATG..TGA)**	None
34	*Croceicoccus naphthovorans* (PQ-2)	1197 **(ATG..TGA)**	1491 **(ATG..TGA)**	Gap (11 bp)	*log* is off-located	570 **(TTG..TAA)**	987 **(GTG..TGA)**	*crtB* is separated from *crtI* by 4 bp	753 **(ATG..TGA)**	525 **(ATG..TGA)**	None
35	*Sphingomonas taxi* (ATCC 55669)	1167 **(ATG..TAA)**	1476 **(ATG..TAG)**	Gap (25 bp)	*log* and *crtB* are off-located	582 **(ATG..TAG**)	951 **(CTG..TAA)**	Overlap (11 bp)	825 **(ATG..TGA)**	468 **(GTG..TGA)**	717 **(ATG..TGA)**
36	*Sphingomonas* sp. (RIT328)	1167 **(ATG..TGA)**	1476 **(ATG..TAG)**	Gap (23 bp)	*log* and *crtB* are off-located	582 **(ATG..TAG)**	948 **(CTG..TGA)**	Overlap (11 bp)	819 **(ATG..TGA)**	468 **(ATG..TGA)**	711 **(ATG..TAG)**
37	*Sphingobium japonicum* (UT26S)	1161 **(ATG..TGA)**	1482 **(ATG..TAA)**	Gap (13 bp)	Gap (9 bp)	582 **(ATG..TGA)**	954 **(ATG..TGA)**	*crtB* is off-located	726 **(ATG..TGA)**	510 **(ATG..TGA)**	None
38	*Sphingomonas* sp. (MM-1)	1185 **(ATG..TGA)**	1479 **(ATG..TGA)**	Overlap (4 bp)	Gap (51 bp)	582 **(ATG..TGA)**	None		None	None	None
39	*Sphingobium baderi* (DE-13)	None	1482 **(ATG..TGA)**		Overlap (4 bp)	582 **(GTG..TAA)**	None		None	None	None

40	*Sphingomonas wittichii* (RW1)	1143 **(ATG..TGA)**	None	*crtY* and *log* overlap by 4 bp		591 **(ATG..TAG)**	None		None	None	None
41	*Sphingopyxis granuli* (TFA)	None	None			582 **(ATG..TGA)**	None		None	None	None
Serial numbers in the first column are the same as in Tables 1 and S1											

Overlaps among *crtB*, *log*, and *crtI*

In bacteria that contain *crt* loci, *crtB* usually occurs immediately downstream of *crtI*, and in some cases their reading frames overlap. Whereas the two ORFs overlapped by 4 bp in strain DSM 22298, they were separated by 4 and 75 bp in strains PQ2 and M41, respectively (Table S2). Surprisingly, in the genomes of 24 strains of *Sphingomonadales*, an unusual ORF was present between *crtI* and *crtB* (Figs. 1 and S1). In nine of the 24 strains, the reading frames of *crtB* and *log* overlapped by 1–4 bp, with 1 bp being the most common overlap (Table S2). In 15 strains where *crtB* and *log* occur together without an overlap of the reading frames, their separation range was 4–363 bp (Figs. 1 and S1; Table S2), indicating that *crtB* could be transcribed independently in some of them. Furthermore, in 11 of the 24 strains, the reading frames of *crtI* and *log* overlapped by 1–4 bp, with 4 bp being the most common overlap (Table S2). In 13 strains where *crtI* and *log* occur together without an overlap of the reading frames, their separation range was 1–299 bp (Figs. 1 and S1; Table S2), indicating that *log* could be transcribed independently in some of them. Interestingly, in 11 of the 24 strains, the reading frame of *crtY* overlapped with that of *crtI*, and the reading frame of *crtI* overlapped with that of *log*. However, only in 3 (*Sphingopyxis terrae* NBRC 15098, *Sphingomonas* sp. Root241, and *Altererythrobacter ishigakiensis* NBRC

107699) of the 24 strains, the reading frame of *crtY/crtI/log* overlapped with that of *crtI/log/crtB*, respectively (thus forming a putative *crtY–crtI–log–crtB* operon).

In the genomes of 6 strains, the *crtY–crtI* and *log–crtB* pairs were separated by 1–2 ORFs (Figs. 1 and S1; Table S2). In two of these strains (*Sphingomonas* sp NIC1 and *Sphingomonas melonis* TY), the reading frames of *log* and *crtB* overlapped by 11 bp, and in the other four, they were separated by 11–61 bp. In the genomes of *Sm. taxi* ATCC 55669 and *Sphingomonas* sp. RIT328, the *log–crtB* pair was located away from the *crt* locus (Figs. 1 and S1) and their reading frames overlapped by 11 bp (Table S2). Furthermore, in the genomes of *Sb. japonicum* UT26S, *Sphingomonas* sp. MM-1, and *Sb. baderi* DE-13, *log* was found immediately downstream of *crtI* (Figs. 1 and S1). Whereas the reading frames of *crtI* and *log* overlapped by 4 bp in *Sb. baderi* DE-13, they were separated by 9 and 51 bp in the other two strains.

Overlaps among *crtZ* and *crtW*

In four strains (JCM 16345, NBRC 107699, HTCC2594, and DSM 9434, which were members of *Erythrobacteraceae*), *crtW* was located immediately upstream of *crtZ* (Figs. 1 and S1), and their reading frames overlapped by 4 bp. Conversely, in two strains (ATCC 55669 and RIT328, which were members of *Sphingomonas*), *crtW* was located immediately downstream of *crtZ* (Figs. 1 and S1). Whereas the reading frames of *crtW* and *crtZ* overlapped by 4 bp in strain ATCC 55669, they were separated by 7 bp in strain RIT328. Therefore, it is likely that there is transcriptional and/or translational coupling of *crtW* and *crtZ* in these strains.

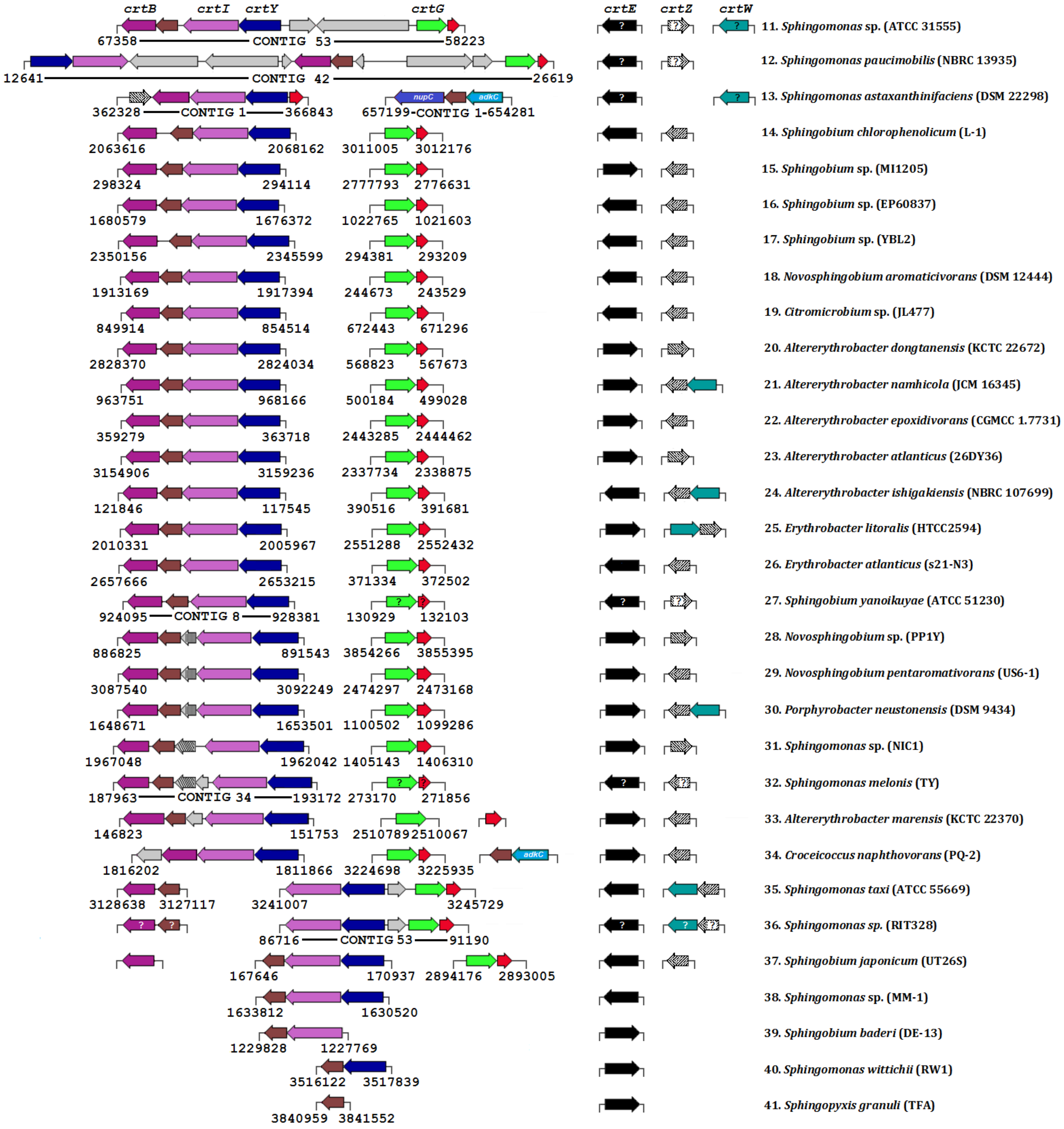

crtB
crtI
crtY
crtG
crtE
crtZ
crtW
67358
CONTIG 53
58223
11. Sphingomonas sp. (ATCC 31555)
12641
CONTIG 42
26619
12. Sphingomonas paucimobilis (NBRC 13935)
362328
CONTIG 1
366843
nupC
adkC
657199-CONTIG 1-654281
13. Sphingomonas astaxanthinifaciens (DSM 22298)
2063616
2068162
3011005
3012176
14. Sphingobium chlorophenolicum (L-1)
298324
294114
2777793
2776631
15. Sphingobium sp. (MI1205)
1680579
1676372
1022765
1021603
16. Sphingobium sp. (EP60837)
2350156
2345599
294381
293209
17. Sphingobium sp. (YBL2)
1913169
1917394
244673
243529
18. Novosphingobium aromaticivorans (DSM 12444)
849914
854514
672443
671296
19. Citromicrobium sp. (JL477)
2828370
2824034
568823
567673
20. Altererythrobacter dongtanensis (KCTC 22672)
963751
968166
500184
499028
21. Altererythrobacter namhicola (JCM 16345)
359279
363718
2443285
2444462
22. Altererythrobacter epoxidivorans (CGMCC 1.7731)
3154906
3159236
2337734
2338875
23. Altererythrobacter atlanticus (26DY36)
121846
117545
390516
391681
24. Altererythrobacter ishigakiensis (NBRC 107699)
2010331
2005967
2551288
2552432
25. Erythrobacter litoralis (HTCC2594)
2657666
2653215
371334
372502
26. Erythrobacter atlanticus (s21-N3)
924095
CONTIG 8
928381
130929
132103
27. Sphingobium yanoikuyae (ATCC 51230)
886825
891543
3854266
3855395
28. Novosphingobium sp. (PP1Y)
3087540
3092249
2474297
2473168
29. Novosphingobium pentaromativorans (US6-1)
1648671
1653501
1100502
1099286
30. Porphyrobacter neustonensis (DSM 9434)
1967048
1962042
1405143
1406310
31. Sphingomonas sp. (NIC1)
187963
CONTIG 34
193172
273170
271856
32. Sphingomonas melonis (TY)
146823
151753
2510789
2510067
33. Altererythrobacter marensis (KCTC 22370)
1816202
1811866
3224698
3225935
adkC
34. Croceicoccus naphthovorans (PQ-2)
3128638
3127117
3241007
3245729
35. Sphingomonas taxi (ATCC 55669)
86716
CONTIG 53
91190
36. Sphingomonas sp. (RIT328)
167646
170937
2894176
2893005
37. Sphingobium japonicum (UT26S)
1633812
1630520
38. Sphingomonas sp. (MM-1)
1229828
1227769
39. Sphingobium baderi (DE-13)
3516122
3517839
40. Sphingomonas wittichii (RW1)
3840959
3841552
41. Sphingopyxis granuli (TFA)

Fig. S1. Comparison of *crt* ORFs/loci in 31 strains of *Sphingomonadales*. Serial numbers of the bacteria are the same as in Table 1. The *crt* ORF that each color-coded or shaded arrow represents is indicated on the top. The brown and red arrows represent ORFs encoding the putative LOG and DUF2141 proteins, respectively. The gray arrows represent the *separating* ORFs that are unlikely to be involved in carotenoid biosynthesis. Numbers at the beginning and end of gene clusters indicate the coordinates within each genome or contig. In the case of *crtE, crtZ,* and *crtW,* arrows pointing to the right indicate that they were located on the *plus* strand, while those pointing to the left indicate that they were located on the *minus* strand. In serial numbers 14, 22, 23, and 28, *crtY* was located on the *minus* strand, but *crtG* was located on the *plus* strand. In serial numbers 15, 16, 17, and 20, *crtY* was located on the *plus* strand, but *crtG* was located on the *minus* strand. In serial numbers 18, 19, 21, 29, 30, and 33, *crtY* and *crtG* were located on the *minus* strand. In serial numbers 24, 25, 26, 31, and 34, *crtY* and *crtG* were located on the *plus* strand. In *Sphingomonas taxi* (serial number 35), *crtY* was located on the *minus* strand, but *crtB* was located on the *plus* strand. In *Sphingobium japonicum* (serial number 37), *crtY* and *crtG* were located on the *minus* strand, but *crtB* was located on the *plus* strand. The uncertainty of the location of some of the ORFs on the *plus* or *minus* strands in the draft genomes of serial numbers 11, 12, 13, 27, 32, and 36 is denoted by a '?' within the respective arrows.

Sphingomonas sp. ATCC 31555 locus tags and sequences

Sphi55_0001, 302 aa, WP_019370787
MASVTHASLALESALRQVAAEIDAQFDRLLEVPDDPRADLYRAMRHAAIGGGKRLRPLLVFATAQLFAVD
KSCAARVATALECIHVYSLIHDDLPAMDDDDMRRGKPTVHKAFDEATAILAGDCLHDLAFELLAHEATHP
DPFVRVDLISELARASGPSGMAGGQMMDLEAEKASFDLATVTRLQAMKTGALISCAVECAAILGRVAPEG
RTGLRGYARDIGLAFQIVDDILDAEGDEALVGKKLGKDGDAGKETFLSLLGIDRAREQARMLVDQAITHL
HAYGPEADLLRDIARFTLERDR

Sphi55_0002, 307 aa, WP_019370947
MRFPGRDAIVATAGESIARGSRSFAAASKLFDPKTRERAWLLYAWCRRCDDIADGQDHGHGMTVVEDPQA
RLAEVRERTAAALAGEWVGDAAFDALRIVAQETGMPHRFAWDLIDGFALDADGWQPTNEDDLLRYCYHVA
GVVGCMMAVVMGVQPEDEAVLDRACDLGLAFQLANIARDIGEDAEAGRCYLPAAWLAEVGLTADNPLADR
SALAILGTRLADRAEAYEISARGGAAALSFRSAWAVLAAADIYGGIARKVRARGAGAWESRTTTSGGEKL
AAIGGAWIAAWKRAPTPRPAGLWTRPR

Sphi55_0003, 193 aa, WP_019370946
MKRIAVYCGSASPANPVYIEAARTLGRTLAERGIGVVYGGGRLGLMGAVADAALEAGGEVIGVIPTLLVN
AEVAHRGLTSLEVCETMHERKARFTELADGFVNLPGGTGTMDELWEAMSWAQLGYHADPIGLLNIAGYYD
KLVEFWEHMGTVGFVRSQHQGLLLVDSTIDGLLAKMADAQPIVPIAQLRRDQL

Sphi55_0004, 492 aa, WP_019370945
MRRAAVIGAGFGGLALAIRLQSAGVDTTLIEARDKPGGRAYYWDRDGFIFDGGPTVITDPDALKELWRLS
GHDISEDVTLKPVTPFYRLSWPDGTTFDYTNDDAVLAGEIAKLDASDIAGYRKFLEYSERVYQEGYVKLG
AKAFLDFKSMLKAAPALMQNQAWRSVYSIVSSFVKNEKLREALSFHTLLVGGNPMATSSIYALIHKLERD
GGVWWAEGGTNRLVAGMIRHFERLGGAVRIGDPVDEILTLGDRVTGLRTKSGFELEVDAVASNADIVHSY
RDLLRNSRSARRTAGRLVNKRFSPSLFVVHFGLRGTFPDIPHHMILFGPRYKGLLDDIYTHGVISEDFSL
YLHHPSASDASMAPEGHSTFYVLAPVPHLGKFPADWSEVGPILEQRILAEVQRRLIPDLADRIVTKFHYA
PTDFRDDLAAHHGSGFSLEPILTQSAWFRVHNRDDAISNLYFVGAGTHPGAGIPGVVGSAKATAELMLGE
MV

Sphi55_0005, 385 aa, WP_019370944
MPSTISCDVAIVGGGLAGGLIALAIRKKKPHCSVRLIEGSARIGGNHLWSFFADDVAPEHRWLAAPLISH
GWSNYDVAFPGHSRTLPARYYSVESEHFDRVVREALGPKGLLLGREAADITPNAVALADGDLVEADGVID
CRGVGDTSALQLGWQKFHGAELALAALNTVQRPLIMDATVPQIDGYRFVYCLPFSPTRMFVEDTYYSDTP
SIDLLATGARIEAYADAAGWQVERVLREEVGSLPVAMGGDHAAYWAAGGRGIAKAGMKAGLFHPMTGYSF
PDAVRTAALIAEEKDYSGEALHQMLYGYSRGLWRKRGFYRMLAAMLFKAAEPEERYRVLERFYRLDAGLI
QRFYAGQSTWGDRVRVLSGKPPVPIGRALSAIWNR

Sphi55_0006, 166 aa, WP_019369087
MSLPTGIALFVVTVLGMELFAYAAHRWIMHGPGWFLHASHHRPRTGNWEWNDLYAAIFAVPSFVLLLGGA
HLGWWTGWAWIGAGIAAYGMIYFGFHDLIVHKRVRHRYIPRSSYMKRIVQAHRLHHAVHTRKGTVSFGFL
YAPRPEVLKAQLRSSGALTQSSTIPD

Sphi55_0007, 265 aa, WP_019370940
MDIALALLTSAVVMTVIVGVRYLAASAGFALATRLRHPGLYAGLDRQIRKEIGWSLASTAIYGIPAGIVA
WGWKAQGWTRIYDDVHAWPLWYLPVSAFLYLFLHDSWFYWTHRAMHMPALFRRYHAVHHASRPPTAWAAM
SFHWGEALSGAIVIPLLVFVIPIHVAALGLVLTIMTVMGVTNHMGWEIFPAWLWRGPVGAWVITASHHQR
HHERYGCNYGLYFRFWDRLCGTDQGLGQFQGTPRARRAGSVPDTGRRIATGDAGD

Sphi55_0008, 128 aa, WP_026009371
*MGGGSPLAT*LEIDFVKLRSSRGLLQVCIAPAPSVFPDCRDGGGAIKRSIPASAGQLRVTGLAPGDYAVAV
IHDANGNGKLDTMMGIPREGFGFSRNPAIGFGPPRFTAAQFPLTGGSDRQEVRMRYLL

Sphi55_0009, 261 aa, WP_037513166
MTRLEAPRDEGRQGRCGIDAQDKRVAQRGRWIAAAIGGAWLAVHVGCIFFWRWSIAAAPLAVFIVLLQAW
LSTGLFIVAHDCMHGSLAPGRPRWNAVVGALCLGAYAGLSYAVLLPKHHAHHAAPGSADDPDFHADAPQR
ALPWFGSFFRTYYTHGQLARITAAAIVYLLLGASLLNIVVFWAVPALLALGQLFLFGTYLPHRHDGRPFA
DKHHARSNTLGPMLSLITCFHFGAYHHEHHLSPGTPWWRLPALRRERLARR

Sphingomonas astaxanthinifaciens DSM 22298 locus tags and sequences

Sasta_0001, 301 aa, WP_029942171
MASVAEATDLLSEAKRIAAGVDALFEQALADRCDGRDRLCAAMRHAGIGGGKRLRPLITVAAARLFAIDE
DRALRAGAAIEAIHVYSLIHDDLPCMDDDDLRRGKPTVHKAFDEATAVLAGDCFHALAFELLADERTHED
PFVRSELVLELAKASGIDGMGGGQAMDLAAEGETLDLGAITRLQQLKTGALIEYAVEAAVVMGRVAPEGR
ARYRGYARNVGLAFQIADDLLDHEGDEEAAGKKLQKDAEAGKATFVSLLGADRARQQAQMLVTQAIEHLH
GHGEEADLLRAIARYAVERDR

Sasta_0002, 312 aa, WP_029940286
MSEADERARLVQAALESISAGSKSFRFASQLFDQQTRERSWLLYSWCRACDDVTDGQTLGHDAERVDDPA
ARLAFLKAKTAEAFAGQPTGLVPFDALRVVAAECAIPQAVAGDHLAGFERDAGGWRPTTTDDLLSYCYQV
AGAVGVMMAHVMGVPPEDEDTLNRAADLGIAFQLANIARDIVDDAKVGRVYLPAEWLAAEGLAGADLADP
AHRPALARLAHRLADMADAYRRSARVGAARLPFRSRWAVLAASGIYGEIATRAAALGPRAWDERITTSKA
EKAALVMEAFWEALWRVRPAPRDGLWTRPAHA

Sasta_0003, 193 aa, WP_037504354
MSHNLKRIAVYCGSAPGNDPAYADAAVALIRAMAGQGIDLVYGGGKLGLMGLIADTMLEAGGEVHGVIPA
ALVDKEVAHLGLTELHQVAGMHERKAKMTELCDAFVCLPGGIGTLDELFEAWTWNALGYHAKPFCLLNVN
GFWNGLGTFMDHVAGAGFLSQARREQLLLAESPAEAINKLDEAARTATQGMVW

Sasta_0004, 487 aa, WP_029940287
MKSAIVIGAGFGGLALAIRLQSAGVKTTIVEARDRPGGRAYVWEKDGHVFDAGPTVITDPDCLQRLWKLS
GHDMSEDVELVPVKPFYRLSWPDGVVFDYTNDDAELKAAMDALNPDDWAGYQRFLAYSAGVYNEGYVKLG
TKAFESLGDMLKAAPALAKYQAWRSVYSIVSSFVKDEHLRQTLSFHTLLVGGNPMTCSSIYALIHKLERD
GGVWFAKGGTNKLIAGMVRQFERIGGTIRLGDPVTAILAENDRVTGVRTASGWSATADAVASNGDVVHSY
GLIEGSDRGQQQVRALKRKRFSPGLFVLHFGLEGTSDLAHHTILFGPRYGGLVNDIYKTGRLATDPSLYI
HHPTITDPSMAPPGCSTFYALAPVPNAGKADVDWAVEGPKYQEVVLDTIAERLIPDVRQRIRTIFHYTPA
DFSADLAAHLGSAFSLEPVLWQSAWFRTHNRDDKLRNLYFVGAGTHPGAGIPGVVGSAEATAGLMLA

Sasta_0005, 380 aa, WP_029940288
MMKRADLVIVGGGLAGGLCALALRRRRPDLRLLLVEPGPSIGGNHLWSFFESDVAPADRWLTDPLIRHRW
PDYEVRFPAHQRHLAEAYQTIESEALDEAVRKALSAEEIVRAEATDLGPTHVTLATGERIEAKAVLDARG
GKAEGLDLGWQKFLGQLLTIPQGHGLTRPIVMDATVDQHDGYRFVYCLPFSPTELFVEDTYYSDGPELDH
DRLRDRIGDYAAAQGWQVADRSREEHGALPVVIGGDFDRLWPAADHVARAGARGGFFHPLTSYSLPDAVR
FAIWLADKATFDARLGAATRARGRRHWRSGAFYRLLTALLFHAAEPGQRYLVLERFYRLSGPLIGRFYAG
MSTGYDKARVLAGKPPVPFFRALRVLRDSL

Sasta_0006, 176 aa, WP_029940285
MSWPAGLALFVSTVLLMEGFAYVLHRFVMHSRLGWNWHESHHRARTGWFERNDLYAVVFALPSILLIWGG
LNGGWGDWATWMGAGVAFYGVIYFGFHDVIVHGRLPHRIVPRSTYFKRIVQAHKLHHAVESRDGAVSFGF
LYAPPVERLKQALQASREAQLRRARGGSTARHEERA

Sasta_0008, 151 aa, WP_051676451
*MTKRFTLAATAGLLLSAPVLMAAQAPQAGAD*LQVHLTGLRSAKGVVHLCLSARPDKFLDCKADKTALART
VPARAAGRLDLGPVRPGTYALLIVHDENQNGKLDMTLGIPREGFGFSNNPAMRPRPPRWEEIRFTMPATP
TTQQIRVRYVL

Sasta_0009, 258 aa, WP_029942176
MAERRRPAYMAPMLSDAQRRRQAMIGLGLAAAITAAFVALHVWSVFFLPLEGAGWWLALPIVAVQTWLSV
GLFIVAHDAMHGSLAPGRPATNLFWGRLTLLLYAGFWLDRLSPKHFDHHRHVGTERDPDFSVDHPTRFWP
WYYAFMRRYFGLREYLVLNALVLAYVLVLKAPLGNLLLFWALPSILSSIQLFYFGTYLPHRHEDAPFADQ
HNARSNDFPVWLSLLTCFHFGYHREHHLSPGTPWWQLPRRRRELALPA

Altererythrobacter ishigakiensis NBRC 107699 locus tags and sequences

Altish_0001, 301 aa, WP_067599965
MAAAIDHSFLKDGLSRVQREVDSVFDALLPVPQDSRARLVEAMRYAAIGGGKRVRPLLLVSTAELFGVNR
DSALRAACAVEAIHVYSLIHDDLPCMDDDALRHGKPTLHLEFDEATAVLAGDSLHALAFEILSDTATSED
PFTRSELILTLGKASGKDGMAGGQMMDMMAEEEDYDLHTITRLQQLKTGALLAASVEMGAIMGHVQPEGR
AHLRAYARDIGLAFQIADDLLDVEGDADKAGKALRKDEGQGKQTFVTLMGAGKAREQARALVDQAIGHLS
VHGDEAKMLAAVARYIVERDR

Altish_0002, 315 aa, WP_067596482
MKRDELVEASRLAIKQGSKSFSAASRLFDRTTRERVWMLYAWCRRCDDLTDAQDMGGELGDQSDIAARVE
HLRQLTANALAGEETGDIAFDAFGQVAREVGLTMEDADAVIAGFQLDAEDWRPRTEADLMRYCWHVAGAV
GVMMAQVMGVKREDDDTLDRACDLGLAFQLANIARDVVEDDAAARCYLPAEWLVEEDIEPGQHTKPHHRR
ELADMAARLVRRMEVHAASSRIGAARLPFRCRWAILSAARIYTAIGREVLAKGPAAWDARVYTSKTQKIG
HAMAAFVEALVNRPPKLDDEPRWRRSDLAENAASA

Altish_0003, 193 aa, WP_067602122
MNRLAVYCGSATPDDPRYIELATEVGRALALREIGVVYGGGRLGLMGAVARGALDAGGEVIGVIPEALFN
SEVANHDCTKLYTVSGMHERKQRFTDLSDGFITIPGGVGTMDELWEAMSWAQLGYHSKPVGLLNTMGFFD
HLIAFNEKMAEVGFVRPAHQNILIHDTTVGGLIDKMAAYEPHTPIFRMKAEDL

Altish_0004, 520 aa, WP_067602119
MNAATPVTPPVRAPQIVDPAKYEGKTACVIGSGFGGMALAIRLQSAGITTTVIEARDKPGGRAYFWEKDG
FTFDGGPTVVTDPPCLKELWEITGHDMAEDVELMKVMPFYRLNWPDGTNFDYSNDEDSLNSEIAKLDPGD
VAGYAEFLKYSEGVHREGYLKLGTVPFLDFKSMLKAAPALVKKQAWRSVYSMVSSYVKSEKLREALSFHT
LLVGGNPMNTSAIYALIHKLEKDGGVWWTRGGTNRLIAGMVRHFERLGGTMRVGDAVTQVHTIGNKASEV
ETESGWKQRFDAVASNADIMHSYRDLLSGSKRGKDYAKSLSRKSFSPGLFVVHFGLEGTWPGIPHHMILF
GPRYKGLLDDIYKQGVLPQDFSIYLHHPTVTDPSMAPKGKSTFYALVPVAHMGKLAVDWDEVGPVLEKRI
LDEIGRRLIPDIHDRIVTKFSYAPKNFAHDLNAHLGSAFSLEPVLWQSAYLRGHNRDDVIDNFYLVGAGT
HPGAGIPGVVGSAKATAGLMLEDLAIKTTA

Altish_0005, 402 aa, WP_067596481
MGGRETDVAIVGGGLAGGLIALALHRAQPDLRFQLIEAGETLGGHHRWSWFDSDLGHEGRALLSPFTYVA
WDQGYEVAFPQYRRPMRAGYRSLASADFDAALREILPERTLSLGQRVCELDANGVTLQSGERINARTVID
CRSVLPSQHLLGGWQIFAGRHFKYDRPHGITQPMIMDASIDQHAPAGNRSAYRFMYVLPLAEDELFFEDT
YYDEARALDEDLLSRRIDQYAADMGFADGVEIGHERGILPVITGGNFKAYRASIEIPGVAMAGARGGFSH
PLTSYTMPIAVENALAIASHAHLSGPLLAHFVRGRANEHWRKTAIYRALGRMLFKATEPERRVNIFQRFY
QLPEELIERFYACHTTLPDQVRILSGKPPVSIPRAIRALASKGKPLLMEKTE

Altish_0006, 166 aa, WP_067596417
MSWWEIIAIVLAALLGMEFFAWYAHKYIMHGWGWEWHRDHHEPHDNTLEKNDLFAVVFGTINAAMYIYGA
LYSDALWWFALGITLYGVIYTLVHDGLVHQRYFRWVPKKGYAKRLVQAHKLHHATIGKEGGVSFGFVFAR
DPVKLKAELKEQRKAGIAKVREADLS

Altish_0007, 240 aa, WP_067596721
MVFGLMFAAVSMTLIVALRYLISSGMFAWITVRLRPGQHDGLKPQIRREIKWSLASAAIYGVPAGLAFWS
WRHLGWTHMYLDPLDYPVWYMPLSALIYLFAHDTWFYWTHRAMHWPVLFRLAHSVHHNSRPPTAWAAMSF
HPIEALSGAIVIPALVLLVPIHMGILGLVLAVMTIMGVTNHMGWEMFPRWLVHSRFGKWVITASHHERHH
EEYQCNYGLYFRFWDKVCGTDRGLTTRFAQ

Altish_0008, 129 aa, WP_067602229
*MNEPPTGT*VTVTITEMRSSEGVVRACMTTNREIFPRCRKDPASHRTVVTASENMTLTFTGVKPGNYAIAL
LHDENDNGKADRVLGMMPKEGFGFSRDAKVQMAPPKFDDAVFEFDGSAAELTIRMRYML

Altish_0009, 255 aa, WP_067596418
MPYRASTVSHPMKLPSRNSQRLLGLVLAALIAGSWLGIHFYAMFVFELSWQAWPQVLLMATLQCWLSVGV
FIVCHDAMHGSLAPGWQRVNSALGAILLFLYAGFAWRKIRDAHFAHHKHTGKDGDPDFDTANPTHFWAWY
WTFFKRYFGWQSLLYVHMVVGIYLFVFGIPFMQIFLLYGAPALLSSLQLFYFGTYRPHRHLGESFADGHN
ARSDNFSTLASLASCFHFGYHLEHHRRPDVPWWALPGARRAGVAA
